# OptoAssay—Light-controlled dynamic bioassay using optogenetic switches

**DOI:** 10.1126/sciadv.adp0911

**Published:** 2024-09-25

**Authors:** Nadine Urban, Maximillian Hörner, Wilfried Weber, Can Dincer

**Affiliations:** ^1^ University of Freiburg, FIT Freiburg Center for Interactive Materials and Bioinspired Technologies, 79110 Freiburg, Germany.; ^2^ University of Freiburg, Department of Microsystems Engineering (IMTEK), 79110 Freiburg, Germany.; ^3^ University of Freiburg, Faculty of Biology and Signalling Research Centres BIOSS and CIBSS, 79104 Freiburg, Germany.; ^4^ INM–Leibniz Institute for New Materials, Campus D2 2, 66123 Saarbrücken, Germany.; ^5^ Saarland University, Department of Materials Science and Engineering, Campus D2 2, 66123 Saarbrücken, Germany.

## Abstract

Circumventing the limitations of current bioassays, we introduce a light-controlled assay, OptoAssay, toward wash- and pump-free point-of-care diagnostics. Extending the capabilities of standard bioassays with light-dependent and reversible interaction of optogenetic switches, OptoAssays enable a bidirectional movement of assay components, only by changing the wavelength of light. Demonstrating exceptional versatility, the OptoAssay showcases its efficacy on various substrates, delivering a dynamic bioassay format. The applicability of the OptoAssay is successfully demonstrated by the calibration of a competitive model assay, resulting in a superior limit of detection of 8 pg ml^−1^, which is beyond those of conventional ELISA tests. In the future, combined with smartphones, OptoAssays could obviate the need for external flow control systems such as pumps or valves and signal readout devices, enabling on-site analysis in resource-limited settings.

## Teaser

A diagnostic tool with light-controlled reagent release and recapture for wash-free assays offers versatile applications.

## INTRODUCTION

Point-of-care (POC) diagnostics is the key player for enabling fast interventions that are crucial for patient’s outcome. For this purpose, POC devices that allow for rapid diagnostics in nonlaboratory settings carried out by untrained personnel have become more commonplace over the past decade. Its importance has even more deepened during the COVID-19 pandemic. One of the most used POC formats are paper-based devices such as lateral flow assays (LFAs), where the sample is added on a cellulose-based test stripe and transported through capillary forces along the stripe. This, however, only allows for a unidirectional sample flow, which fundamentally limits the flexibility in assay design and sample processing (*1*, *2*). On the other hand, other POC platforms offering bidirectional microfluidics suffer from expensive and bulky pumps or flow control systems, also requiring an additional energy source, which complicates their use in resource-limited settings (*3*, *4*).

**Fig. 1.**
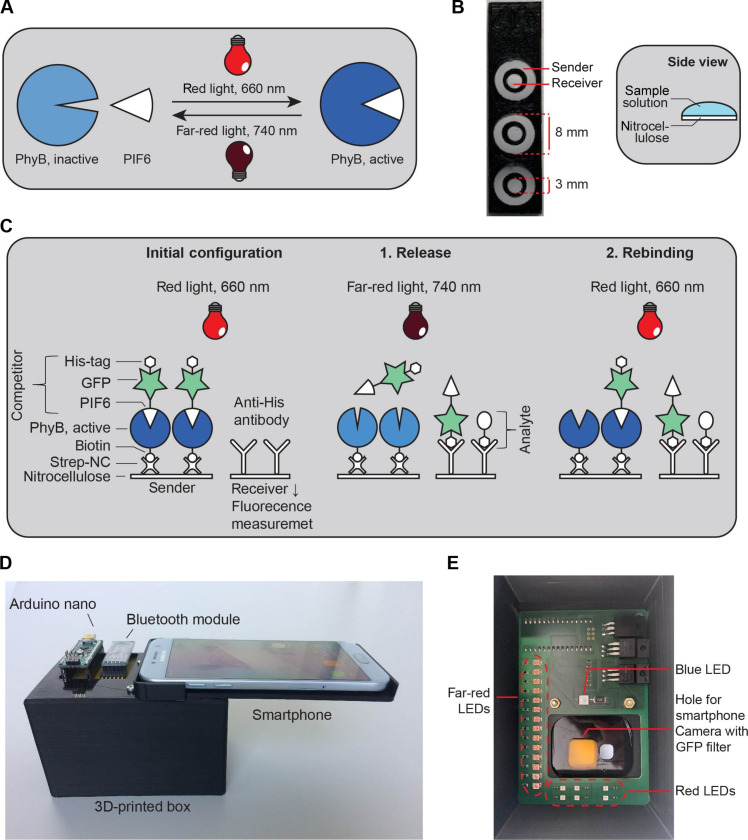
Assay setup. (**A**) Scheme describing the basics of PhyB and PIF6 interaction. PhyB is transformed in the active state by illumination with red light (660 nm) where it interacts with PIF6. By exposure to far-red light (740 nm), PhyB is converted to the inactive form where it cannot interact with PIF6. (**B**) Image of the three-dimensionally (3D) printed sample holder with the laser-cut sender and receiver membranes. The circle-shaped receiver area is enclosed by a larger ring-shaped sender area. Both zones are connected by sample solution on top of the membranes, as illustrated with the side view scheme. (**C**) Working principle of the OptoAssay. In the initial state, active PhyB is immobilized via streptavidin-NC (Strep-NC) on the nitrocellulose sender area, while the competitor complex [green fluorescent protein (GFP)–PIF6-His_6_] is associated with PhyB during red light illumination. The receiver area is coated with anti–His-tag antibodies. At the second step, the assay is illuminated with far-red light; the competitor is released from inactive PhyB, while at the same time, the analyte (His-tagged protein) is added. The analyte and the competitor compete now for binding to the antibodies on the receiver area. The final step comprises illumination with red light to reassociate the unbound competitor to PhyB on the sender area. (**D**) 3D-printed PhotoBox that allows for sample illumination and signal readout for the envisioned POC scenario. A smartphone is placed on top of the box and connected via a Bluetooth module to an Arduino Nano to switch on and off illumination. Through a hole in the box, images can be taken with the smartphone camera. (**E**) PhotoBox from the inside: light-emitting diodes (LEDs) for far-red and red light illumination, blue light LED for GFP excitation, and GFP emission filter.

To circumvent this restraint, we present the proof of principle of a light-controlled dynamic bioassay (OptoAssay). OptoAssays allow for bidirectional, light-induced movement of biomolecules, enabling wash-free signal readout of bioassays. For this purpose, the biomolecules fused to a phytochrome-interacting factor 6 (PIF6) can be released from and rebind to the plant photoreceptor phytochrome B (PhyB) by exposure to far-red or red light, respectively, thereby circumventing the need for external flow control devices. PhyB exhibits two distinct light-absorbing conformations (Fig. 1A): a red light–absorbing *P*_r_ (red) state with λ_max_ ~ 663 nm and a far-red light–absorbing, biologically active, *P*_fr_ (far red) state with λ_max_ ~ 726 nm (*5*). In the active state, PhyB can bind its PIF, while in the inactive state, this interaction is reversed (*5*, *6*). The timescale of switching between the two states is in the millisecond range (*7*, *8*). Up to date, this switching behavior of the PhyB/PIF systems has been used in various applications to control, for example, gene expression (*9*) or cell signaling (*10*), or to create biohybrid materials (*11*, *12*).

Here, we apply optogenetic switches for realizing the first light-controlled dynamic bioassay on a nitrocellulose substrate and successfully demonstrate its proof of principle by performing a calibration curve of a competitive model assay of a hexahistidine-tag (referred to as His_6_-tag) as analyte. Besides, we show the general versatility of the assay components on other substrates, namely, agarose beads and poly(methyl methacrylate) (PMMA), also usually used for building POC devices.

## RESULTS

The concept of OptoAssay consists of two distinct areas (Fig. 1B): a smaller receiver area where the signal readout is performed is placed in the middle of the sender area into a notch so that the areas are not directly connected. These areas are later bridged by adding sample solution to both sides so that the assay components can diffuse from one area to the other. In this work, the OptoAssay is carried out as an immunoassay using antibodies. Herein, a competitive assay format was chosen, in which a competitor and the analyte in the sample compete for binding to the detection antibody. The sender area (Fig. 1C) is treated with streptavidin (Strep) so that the biotinylated photoreceptor PhyB can be immobilized via the biotin-Strep interaction. Here, PIF6 can bind to PhyB or be released depending on the wavelength applied. On the other hand, antibodies directed against the molecule of interest are immobilized on the receiver area.

For the proof-of-principle assay, we use a His_6_-tagged protein (see Materials and Methods) as model analyte and a His_6_-tagged PIF6 as competitor, both of which are recognized by the anti–His-tag antibody immobilized in the receiver area. The signal readout is performed by measuring the fluorescence intensity of the competitor complex, His_6_-tagged PIF6, to which green fluorescent protein (GFP) is fused. For the initial OptoAssay configuration, the competitor complex is attached to PhyB on the sender area during red light exposure. The assay procedure itself comprises two steps (Fig. 1C): First, the analyte is added, and then, far-red light illumination is used to release the competitor complex from PhyB; thus, both the analyte and the competitor can compete for binding to the antibody immobilized on the receiver area. Second, red light is applied, which leads to conformational change in PhyB so that it can bind to PIF6 again. This enables the reassociation of competitor complexes (His_6_-tagged PIF6) that are not bound to the anti- His_6_ antibodies with PhyB on the sender area, resulting in the removal of the unbound competitor from the receiver area. Because of the competitive assay format, the signal measured on the receiver area is inversely proportional to the concentration of the analyte. To enable POC testing using the OptoAssay, a three dimensionally (3D) printed PhotoBox that allows for illumination with red and far-red light, as well as a blue light for GFP fluorescence excitation, has been built (Fig. 1, D and E) (*13*). The illumination can be controlled with a smartphone that is linked via a Bluetooth module to the electronics of the PhotoBox. For the fluorescence detection, an emission filter is placed at the front of the smartphone camera. Images of the OptoAssay results can then be read out easily, evaluated, and further transmitted to, for example, medical facilities via smartphone (see movie S1).

First, we confirmed the functionality of our assay components and determined key kinetic parameters of the PhyB/PIF system (see movie S2). We used a fibrin hydrogel as a matrix where we incorporated NeutrAvidin (nAv) and, subsequently, the biotinylated PhyB, which was associated with GFP-PIF6 during 660-nm illumination. We then illuminated the hydrogel spatially resolved with 740-nm light (<4 s) using a confocal microscope to locally release GFP-PIF6 (fig. S1). Afterward, we globally illuminated the hydrogel with a 740-nm light to equally distribute GFP-PIF6 and reassociated PIF6 to PhyB under global 660-nm illumination. We repeated this cycle three times and determined the half-life of the GFP-PIF6 release as 2.91 ± 0.27 s. The regeneration time of the PhyB/PIF6 interaction upon 660-nm illumination cannot be directly determined from these data. However, the time between the start of the global 660-nm and the localized 740-nm illumination, which was 57 and 53 s, can be regarded as the upper limit.

As substrate material for immobilization of assay components, however, we chose nitrocellulose because it is widely applied for LFA devices because of its nonspecific affinity to proteins (*14*, *15*) and does not need any preparation in comparison to the hydrogel. We first investigated the general functionality of the optogenetic system on a nitrocellulose substrate by testing a spatially resolved release of PIF from the substrate (fig. S2). In addition, we were interested whether the immobilization of the photoreceptor PhyB through a biotin-binding protein, here, nAv, is necessary because nitrocellulose has the ability to bind proteins nonspecifically. Therefore, we incubated one membrane with nAv before PhyB immobilization, and one membrane was left untreated before PhyB immobilization. After GFP-PIF6 was added during red light illumination, the membranes were illuminated at 740 nm through a photomask. The results (fig. S2B) successfully demonstrate a spatially resolved release of GFP-PIF6 from the substrate. It also shows that PhyB immobilization without nAv leads to lower fluorescence intensity in general, which might indicate lower functionalization of nitrocellulose or decrease in functional PhyB. Conclusively, we used a biotin-binding protein for PhyB immobilization for subsequent experiments.

Because the PhyB/PIF interaction plays a crucial role in our experimental setup, we tested whether a competitor complex containing two PIF6 proteins shows better performance in terms of leakiness, i.e., fewer unspecific release during red light illumination due to increased avidity. Therefore, the release of competitor complexes containing one or two PIF proteins was measured over a time range of 80 min. In addition, to determine the unspecific release of the nitrocellulose membrane itself, the release of a biotinylated version of GFP-PIF6, immobilized directly on nAv, was determined. According to our findings (fig. S3), the double PIF version has no advantages over the single version regarding the unspecific release (red light, 660 nm). The release of competitor molecules can be attributed to the unspecific release of the nitrocellulose substrate itself, as the biotin-GFP-PIF6 version shows similar values than the two other nonreleased samples. However, far-red light illumination (740 nm) yielded higher release for the single PIF version, which is why we conducted all following experiments with this competitor version.

Moreover, we designed a measurement setup (Fig. 1B), including sender and receiver areas, for the OptoAssay. For this purpose, we used a laser cutter to create evenly sized round nitrocellulose structures (receiver Ø, 3 mm; sender-outer Ø, 8 mm; and sender-inner Ø, 4.4 mm). In addition, we 3D-printed frames to put these OptoAssay structures in place for easier handling.

**Fig. 2.**
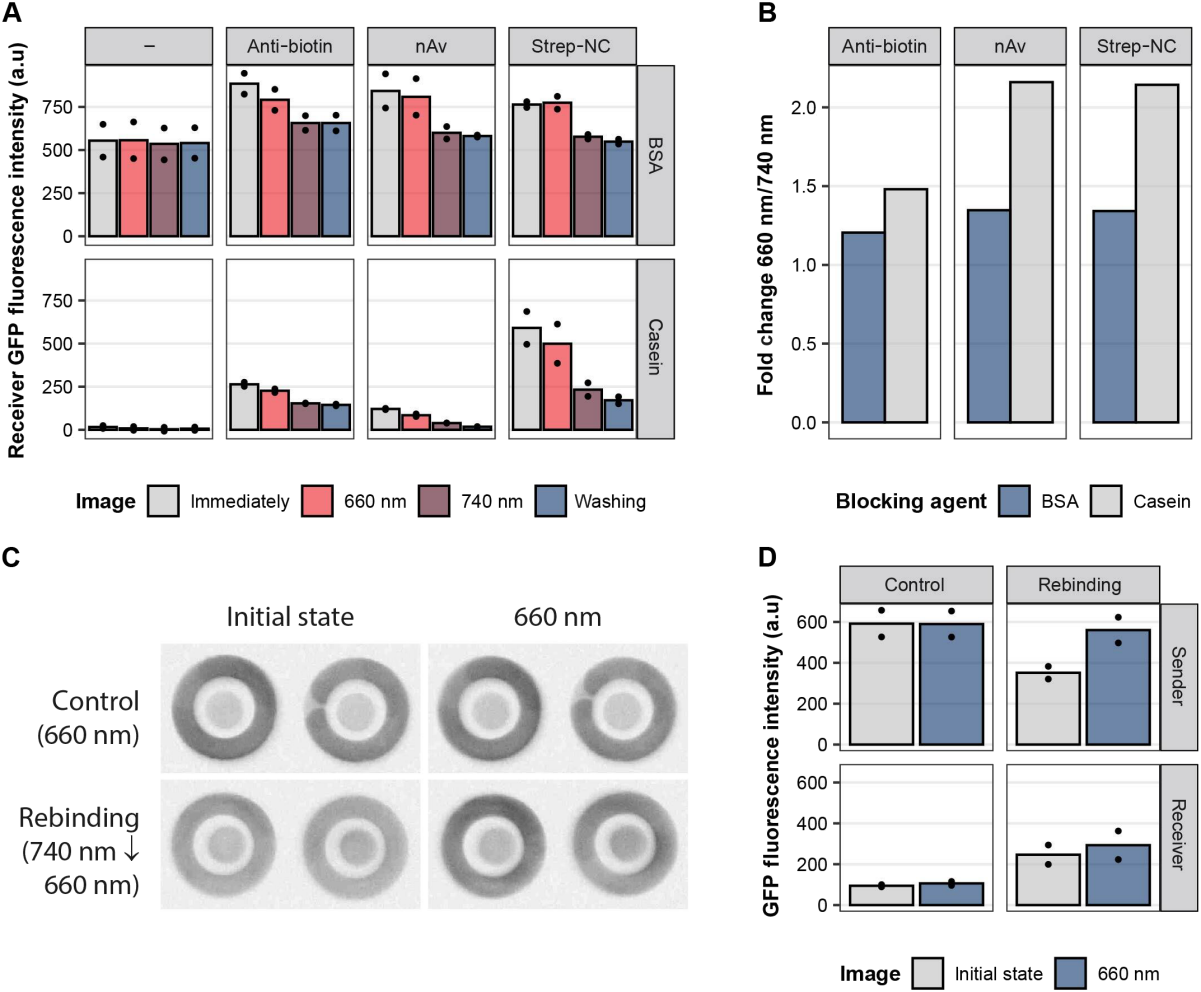
Establishing the assay setup on nitrocellulose. (**A**) Testing different biotin-binding proteins in combination with different blocking agents. Nitrocellulose membranes were coated with anti-biotin antibody, nAv, or Strep-NC, respectively. The membranes were blocked with either BSA or casein. For the negative control (−), untreated membranes were blocked with the respective blocking agent only. Samples were then incubated with PhyB-biotin and, subsequently, with GFP-PIF6-His_6_. Images were taken immediately after sample preparation, after 660-nm incubation, after 740-nm incubation, and after the samples were washed with wash buffer. Data represent the means of *n* = 2 samples; dots represent single values. (**B**) Fold change of the fluorescence intensity of the 660- to 740-nm image from (A). Fold change was calculated by first subtracting the background value (−) for each biotin-binding protein/blocking agent combination from the respective 660- or 740-nm value. Then, 660-nm values were divided by 740-nm values. (**C**) Reassociation of GFP-PIF6-His_6_ to PhyB. Fluorescence images at the initial state and after 660-nm illumination. Sender membranes (outer ring) were incubated with Strep-NC and then blocked with casein. Next, biotinylated PhyB was immobilized. The competitor complex (His-GFP-PIF6) was bound to PhyB under red light illumination. The receiver membranes (inner circle) were coated with anti–His-tag antibody, dried, and subsequently blocked with casein. Control samples were kept at 660 nm during the entire time of the experiment. The rebinding samples were first illuminated with a 740-nm light (initial state) and then exposed to a 660-nm light. Images were taken at either 660- or 740-nm illumination and after subsequent illumination with 660 nm. (**D**) Sender and receiver membrane fluorescence intensity values quantified from (C). Columns represent the means of *n* = 2 samples; points represent the single values. a.u., arbitrary units.

Next, we examined different immobilization strategies; in addition to nAv that was used for immobilization so far, an anti-biotin antibody and Strep-NC, an engineered version of Strep that has better nitrocellulose binding properties than the wild-type version, were tested (*16*). After adding the respective immobilization protein (nAv, Strep-NC, or anti-biotin antibody) to the nitrocellulose membranes (Ø, 3 mm) and drying overnight (or leaving membranes without coating as a control), either bovine serum albumin (BSA) or casein was used as a blocking agent before introducing biotinylated PhyB. Then, GFP-PIF6-His_6_ was added and incubated for 10 min during red light exposure to bind to PhyB. Afterward, unbound GFP-PIF6-His_6_ was removed, and the membranes were washed before assembly onto the 3D-printed frames and addition of buffer. Images of the membranes were taken (i) immediately after assembly, (ii) after illumination with a 660-nm light for 10 min, (iii) after subsequent 10-min illumination with a 740-nm light, and (iv) after washing the membranes at the end. The fluorescence signals (Fig. 2A) clearly show that BSA blocking leads to higher background signal compared to casein blocking. In the case of BSA blocking, the background signal (−), i.e., a blocked membrane plus GFP-PIF6-His_6_, is, on average, over the four images, 66 times higher than for casein blocking. When looking at the different biotin-binding proteins, the signal height for BSA blocking does not vary much, while it does for casein blocking: Strep-NC clearly leads to the highest signal intensity between the three biotin-binding proteins (nAv, Strep-NC, and anti-biotin antibody). Because we are interested in a high dynamic range between the initial state (660 nm) and release of the competitor (740 nm) for the OptoAssay, we calculated the fold change of intensity for 660 and 740 nm for each protein and blocking agent combination after subtracting the background value (−) for the respective blocking agent from each value (Fig. 2B). As expected, the high unspecific binding and, thus, high background signal with BSA blocking led to a lower fold change for all biotin-binding proteins compared to casein blocking. For casein blocking, although nAv, on average, showed 6.6 times lower signal compared to Strep-NC, the fold change of 2.1 was the same as for nAv and Strep-NC. We lastly decided to continue using Strep-NC for biotin-PhyB immobilization because of its higher binding capacity.

After these improvements, we tested the complete OptoAssay configuration meaning to release and rebind the competitor without introducing a sample spiked with the analyte. For this reason, we immobilized PhyB and GFP-PIF6-His_6_ on the sender membrane (see Materials and Methods) and anti-His antibodies on the receiver membrane. The membranes, first, were illuminated with a 740-nm light where the competitor GFP-PIF6-His_6_ was released and could bind to the receiver followed by illumination with a 660-nm light to reassociate unbound GFP-PIF6-His_6_ to the sender. The control membranes were constantly kept at 660 nm. When observing the sender membranes in their initial state (Fig. 1C), it became apparent to the naked eye that the fluorescence intensity differed between the rebinding and control samples. The rebinding condition, illuminated at 740 nm, exhibited lower fluorescence compared to the control sample, which remained under constant illumination at 660 nm.

In addition, the reassociation for the rebinding condition after applying a 660-nm light was clearly visible, suggesting a successful light-dependent release and rebinding of the competitor complex from and to the nitrocellulose substrate. This could also be verified by quantifying the intensity values (Fig. 2D): For rebinding, after applying a 660-nm light, the fluorescence intensity on the sender membranes was almost as high as for the control samples. The quantified intensity values of the receiver areas show that the membranes had 2.7 and 2.8 times higher intensity after rebinding, for the initial state and the 660-nm image, respectively, than the control. This demonstrated again the functionality of the system where the competitor could reversibly be transferred to the receiver membrane in a light-mediated manner.

Having successfully demonstrated the general functionality on a nitrocellulose substrate, we tested the performance of the optogenetic components on other substrates. As an alternative planar substrate, we used PMMA, which is low-priced and, thus, convenient for mass production of a potential POC device. A preliminary experiment (fig. S4) showed that nAv directly absorbed on untreated PMMA, which circumvented the need for surface functionalization. Subsequently, we immobilized biotinylated PhyB on the nAv-coated surface. With this setup, we could illustrate a spatially resolved release of the competitor complex from a PMMA substrate (fig. S5). As the next substrate, we evaluated beads that are often used as solid-phase materials in bioassay applications in clinical and POC diagnostics (*17*, *18*). In this setting, we first verified the functionality of the optogenetic system on a bead-based platform where nAv-functionalized agarose beads were used to immobilize biotinylated PhyB. We established the reversibility of releasing and rebinding the PIF6 molecule as a competitor from PhyB, following the procedure outlined in (fig. S6) (*11*). A substantial increase in supernatant fluorescence intensity of 7- and 15-fold for the higher (2.9 mg ml^−1^) and lower (0.029 mg ml^−1^) initial concentration of mCh-PIF6, respectively, when comparing the release sample (740 nm) to the control (660 nm) was observable. The reversibility is shown by the reassociation of previously released mCh-PIF6 molecules (switching back from 740 to 660 nm), indicated by a 9 or 51% decrease in supernatant fluorescence intensity for the upper and lower initial concentration of mCh-PIF6, respectively. Once more, we demonstrated light-dependent release and partial rebinding of competitor molecules onto and from the substrate, indicating the probable versatility of the OptoAssay across diverse assay formats.

At the next step, we implemented the previously optimized parameters for the nitrocellulose substrate and performed a calibration curve of a competitive OptoAssay where different concentrations of a His_6_-tagged analyte were introduced. Fluorescence intensity values of the membranes were measured in the following order: (i) after 15-min release and (ii) 30-min release with 660 nm, (iii) subsequent 40-min rebinding with 740 nm, and (iv) a final manual washing where the buffer was exchanged. The normalized intensity values (*I*/*I*_0_) were obtained by dividing *I*_0_, the value where no analyte was present, by the values where analyte was present (*I*). We applied a four-parametric logistic fit to calculate the limit of detection (LOD) (*19*, *20*). For release and rebinding conditions, the LOD ranges between 1 and 12 pg ml^−1^, with 8 pg ml^−1^ for rebinding, while it was roughly 1000 times higher for the washing condition.

Looking at the fitted curves, it is observable that as the experiment progresses, the curve becomes more sigmoidal in shape, which describes the typical response between the relationship of concentration and signal for immunoassays (*19*, *20*). While the curve for 15-min release rather describes the shape of a straight line, a more curved shape can be observed after 30-min release. After rebinding, i.e., removing of unbound components, an improvement in the sigmoidal shape is clearly visible. This indicates that rebinding and, therefore, removing background noise, allows for a more specific, reliable and controlled response to the analyte concentration in comparison to release only. The curve shape is retained after the final washing step, however more flattened in comparison to rebinding, which, therefore, in combination with the higher standard deviations (SDs) of the data points, results in a much higher LOD. The mean coefficient of variation is the lowest for rebinding with 9.8%, while it is 18.3, 12.8, and 13.9% for 15-min release, 30-min release, and washing, respectively.

**Fig. 3.**
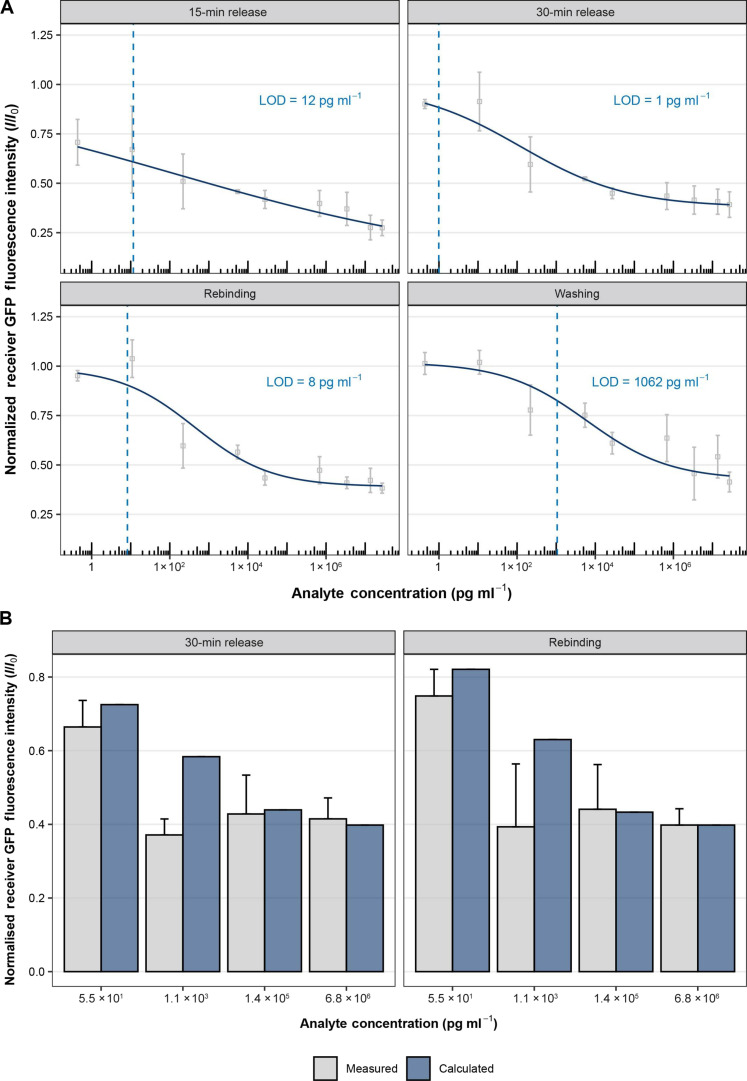
Assay calibration and spike-and-recovery measurements. (**A**) Assay calibration. The competitor complex (His-GFP-PIF6) was released via 740-nm illumination, while the analyte (GyrB-His) in different concentrations ranging from 2.73 × 10^7^ to 4.36 × 10 ^–1^ pg ml^−1^ was introduced to the assay. GFP fluorescence intensity of the receiver areas was measured after 15 and 30 min of 740-nm illumination, after rebinding with 660 nm and after manually washing the samples. For the normalization, intensity values from analyte samples were divided by a negative control. (*I*/*I*_0_). Gray dots represent the means of *n* = 3 samples; error bars represent SDs. The continuous dark blue lines illustrate fitted values that were obtained by applying a four-parametric logistic fit to the data. The dotted light blue lines indicate the LOD. (**B**) Spike-and-recovery measurements. Different analyte concentrations (55, 1.1 × 10^3^, 1.4 × 10^5^, and 6.8 × 10^6^ pg ml^−1^) were introduced. The samples were first illuminated for 30 min with far-red light (30-min release) and 40 min with red light (Rebinding). For the normalization, intensity values from analyte samples were divided by a negative control (*I*/*I*_0_). The gray bars represent the means of *n* = 3 samples at the given analyte concentration; error bars represent SDs. The blue bars represent the calculated values using the previously established four-parametric logistic fit for the given concentration.

Last, we wanted to validate the previously established model by performing spike-and-recovery measurements. For that reason, we carried out the assay with four different analyte concentrations that were different from the ones from the calibration curve and compared the measured values to the model-derived values after 30-min release and rebinding (Fig. 3B). The deviation of the measured to the calculated values range from <0.1 to 37.6%, where we see the highest difference of 36.4 and 37.6% at an analyte concentration of 1.1 × 10^3^ pg ml^−1^ for 30-min release and rebinding, respectively, which can be explained by the proximity to the infliction point. The observed values vary the lowest from the predicted values at the highest analyte concentration of 6.8 × 10^6^ pg ml^−1^ with 4.2 and < 0.1% and the second highest, 1.4 × 10^5^ pg ml^−1^, with 2.5 and 1.8%. A variation of 8.3 and 8.8% was observed for the highest concentration of 55 pg ml^−1^. These data demonstrate that the OptoAssay provides promising results across a wide range of analyte concentrations, showing its potential for POC diagnostics.

## DISCUSSION

In this work, we have successfully demonstrated the proof of principle of OptoAssay that enabled, in contrast to conventional POC tests such as LFAs, an optically driven, bidirectional sample flow without any pump or flow control systems for a wash-free signal readout. Through a photoreceptor that can light-dependently interact with a binding partner, a competitor molecule can be released and later removed from the detection area by subsequently applying far-red and red light. With a model assay, we performed a calibration curve and statistically calculate an LOD of 8 pg ml^−1^, which was lower than the typical LODs of standard competitive enzyme-linked immunosorbent assays (ELISAs) ranging from 0.1 to 10 ng ml^−1^, depending on the analyte (*21*–*23*). Although the lowest LOD was not reached with the rebinding condition, the shape of the fitted curve shows that rebinding has the most accurate representation of data in terms of the expected curve shape, given the assay format and the lowest variation within the samples. Thus, it indicates that light-triggered rebinding yields a faster establishment of equilibrium and an improved signal-to-noise ratio.

Our experimental results also demonstrate that the values show a reasonable correspondence with the calculated calibration curve. Specifically, the deviations between the measured and calculated values ranged from less than 0.1 to 37.6%. Notably, the highest discrepancies were observed at an analyte concentration of 1.1 × 10^3^ pg ml^−1^ for 30 min of release and rebinding, respectively. This concentration corresponds to the range around the inflection point of the calibration curve, which is inherently associated with greater variability because of the nonlinear response characteristics of the system at this stage, and slight variations in the system’s response are amplified (*24*). Therefore, small deviations of the theoretical concentration compared to the actual concentration lead to large deviations of the predicted compared to the measured value. Conversely, at the highest and lowest analyte concentrations, the variations were minimal. Similarly, low deviations could be observed at the second highest concentration. These findings emphasize the precision of the model at higher concentrations where the calibration curve is more stable. Overall, these results validate our model’s predictive capability, and the observed variations remained within acceptable limits.

Although other photoswitchable assays already exist (*25*, *26*), they only allowed for detection of specific molecules, whereas the OptoAssay can universally be applied by fusing the molecule of interest to PIF6. For example, integrating a zz domain (*27*) for immunoglobulin G binding could make this format compatible with antibody-based bioassays. The PhyB/PIF system shows functionality in different matrices such as BSA, fetal bovine serum, and various cell culture media (*11*, *28*), making it ideal for the application in biological specimen.

The system could also be further expanded by using or combining different optogenetic switches that respond to distinct wavelengths. For instance, the blue light receptor cryptochrome 2 (Cry2) that forms heterodimers with its interaction partner cryptochrome-interacting basic helix-loop-helix (CIB1), or another system, improved light-induced dimer (iLID), which comprises of the blue light receptor light oxygen voltage 2 domain of *Avena sativa* phototropin 1-SsrA (AsLOV2) and its binding partner stringent starvation protein B, could be used (*29*). However, both photoreceptors only enable an active associating with its interaction partner; the dissociation is usually a slower passive event induced by the absence of blue light. Therefore, the OptoAssay workflow might need to be adapted accordingly to use different or the same photoreceptors for measuring multiple analytes or realizing complicated functions (such as mixing or relocation of biomolecules).

Using a 3D-printed PhotoBox along with a smartphone, the OptoAssay introduced could be easily used in on-site applications. Herein, we extended the PhotoBox developed in a previous work (*13*) with a blue excitation light (460 to 470 nm) and emission filter that allowed for GFP detection using a smartphone. Using this setup, automated sample illumination and evaluation of the results via a smartphone app can be realized for user-friendly operation.

Another issue that must be addressed is the rebinding efficiency and time of the competitor molecules used for the OptoAssay, which is, at the moment, mainly limited by the diffusion of the biomolecules used. Herein, there is one important factor: the overall distance that the molecules have to travel between the two areas (i.e., sender and receiver zones). In our initial experiment, we determined the half-life of 2.91 ± 0.27 s for the PIF6 dissociation from PhyB, which is using a fibrin hydrogel as immobilization substrate. However, here, the diameter of the observed area was only 12 μm, showing that the dimensions of the assay structures can have a substantial impact on the operation time. To decrease the distance in our current OptoAssay setup and, therefore, the operation time, which is around 70 min (30-min release along with 40-min rebinding) at the moment, smaller assay areas could be designed and fabricated by micro/nanofabrication techniques.

The OptoAssay system’s complexity and reagent requirements may affect reproducibility, repeatability, accuracy, and precision. These issues can be mitigated through standardization, optimized protocols, and automation to reduce human error. Calibrated light sources and real-time monitoring address light power fluctuations and thereby maintain consistent conditions. Future improvements may include fully automated systems, simplified reagent kits, and user-friendly interfaces and devices. These enhancements will streamline processes, improve reliability, and ensure the practical application of OptoAssays in diverse and resource-limited settings.

With regard to the applicability of the OptoAssay in a POC setting, the current cost of our bioassay format is around €5.6 per sample (Table 1), mainly because of high Strep-NC and anti-His antibody expenses. Strep-NC could be avoided by directly coupling PhyB to the matrix, whereas costs for the antibody binding the analyte are needed for any assay format. Further, mass production could significantly lower these costs through economies of scale, bulk purchasing, and optimized production processes. Technological advancements and streamlined manufacturing can further reduce expenses. Lower production costs will enhance market competitiveness and accessibility, especially for POC devices. Strategic partnerships and funding can also offset initial costs. Thus, with scaling, our bioassay format can achieve cost efficiency, maintaining high quality while being economically viable and widely adoptable. Furthermore, reducing the dimensions as mentioned above, could, in addition to decreasing the assay operation time, lower the cost, as fewer amounts of reagents would be needed.

**Table 1.** Cost estimation of the OptoAssay for one sample.

Materials	Cost per sample
Nitrocellulose	€0.016
Strep-NC	€2.779
Casein	€0.373
PhyB	€0.432
PIF6	€0.008
Anti-His antibody	€1.962
Sum	**€5.569**

In conclusion, OptoAssay provides the basis for an innovative and dynamic bioassay format independent of assay type and biomolecules used, such as antibodies, proteins, or nucleic acids. Through its light-dependent, two-way switch, it can extend already existing POC devices and could pave the way for new classes of diagnostic devices, extending the capabilities of state-of-the-art POC tools.

## MATERIALS AND METHODS

### Plasmids and oligos

The plasmids generated with Gibson cloning (*30*) and used in this study are described in table S7.

### Protein production and purification

#### PhyB constructs

PhyB-AviTag was produced by high–cell density fermentation, as described previously (*31*). PhyB-AviTag (pMH1105) bacterial pellets, stored at −80°C, were dissolved in Ni lysis buffer [50 mM NaH_2_PO_4_, 300 mM NaCl, and 10 mM imidazole (pH 8.0)], with 10 ml of buffer per 1 g of bacterial pellet. The suspension was lysed using a French press (APV 2000, APV Manufacturing) running three cycles at 1000 bar. The lysate was centrifuged at 17,000*g* for 30 min at 4°C; then, the supernatant was transferred to a new vessel and centrifuged again for 30 min. The protein was purified using a Ni-NTA Superflow column (QIAGEN, Netherlands, 30761) on an ÄKTAxpress fast protein liquid chromatography system (GE Healthcare, USA). The lysate was loaded on the column, which was afterward washed with 12 column volumes of Ni wash buffer [50 mM NaH_2_PO_4_, 300 mM NaCl, 20 mM imidazole, and 0.5 mM tris(2-carboxyethyl)phosphine (TCEP) (pH 8.0)]. The protein was eluted in 6 column volumes of Ni elution buffer [50 mM NaH_2_PO_4_, 300 mM NaCl, 250 mM imidazole, and 0.5 mM TCEP (pH 8.0)]. The eluted protein was stored at 4°C or frozen at −80°C.

Before freezing, the protein was concentrated using centrifugal concentrators (Corning Inc., USA, 431491), and the buffer was exchanged to Dulbecco’s phosphate-buffered saline [DPBS; 2.7 mM KCl, 1.5 mM KH_2_PO_4_, 136.9 mM NaCl, and 8.9 mM Na_2_HPO_4_ × 7H_2_O (pH 7.0)] with a 10-ml dextran desalting column (Thermo Fisher Scientific, USA, 431489). The protein was aliquoted, shock frozen in liquid nitrogen, and stored at −80°C.

#### PIF6 constructs and analyte protein (GyrB-6His)

For protein production, the corresponding plasmid (table S7) was transformed into chemically competent *Escherichia coli* BL21 Star (DE3) (Thermo Fisher Scientific, USA, C601003). The bacteria were cultured in LB medium supplemented with ampicillin (100 μg ml^−1^) and were grown overnight at 37°C and 150 rpm. The overnight culture was transferred to new LB medium supplemented with ampicillin to reach an OD_600_ (optical density at 600 nm) of 0.15 and cultured at 30°C and 150 rpm. Upon reaching an OD_600_ of 0.5 to 0.8, protein production was induced by 1 mM isopropyl-β-d-thiogalactopyranoside and transferred to 18°C for 20 hours and shaking at 150 rpm. Cells were subsequently harvested by centrifugation at 6500*g* for 10 min, resuspended in lysis buffer, and disrupted using a French press at 1000 bar or by sonication (Bandelin Sonopuls HD3100, Bandelin, Germany). Lysates were clarified by centrifugation at 30,000*g* for 30 min and loaded on a Ni-NTA Superflow column (QIAGEN, Netherlands, 30761). After washing with each 20 column volumes of lysis and wash buffer [50 mM NaH_2_PO_4_, 300 mM NaCl, and 20 mM imidazole (pH 8.0)], purified proteins were eluted in 5 column volumes of Ni elution buffer [50 mM NaH_2_PO_4_, 300 mM NaCl, and 250 mM imidazole (pH 8.0)]. Last, the proteins were quantified by measuring the GFP fluorescence (excitation wavelength, 488 nm, and emission wavelength, 530 nm) or by Bradford assay (Bio-Rad, USA, 5000006).

### Illumination experiments

#### OptoAssay

Nitrocellulose (Thermo Fisher Scientific, USA, 88018) was cut into the respective sender and receiver shapes using a laser cutter (Universal Laser Systems, USA, PLS6.150D). The receiver membranes were marked on the backside with a pencil and put on Parafilm in a petri dish facing the top side up. Then, 2 μl of anti-His antibody (0.2 mg ml^−1^ in deionized water; Merck, Germany, NOVG70796-3) were added and incubated for 1.5 to 2 hours at 37°C, subsequently put in 24-well plates, and blocked with casein (1.375%; Fitzgerald Industries, USA, 85R-108_1l) for 5 min. The membranes were washed twice with DPBS for 5 min and stored in PBS until it was further used.

The sender membranes were also marked on the backside and put on Parafilm in a petri dish; then, 7 μl of Strep-NC (1.42 mg ml^−1^ in DPBS; IPOC, Canada, RP-9000) was evenly distributed on a nitrocellulose sender membrane and dried at 37°C for 1.5 to 2 hours. The membranes were then put in a 24-well plate and blocked with casein as previously described. Afterward, 120 μl of PhyB (pMH1105) with a final concentration of 0.5 mg ml^−1^ in DPBS with 10 mM TCEP were added to each membrane and incubated for 30 min in a shaker while covered with aluminum foil. Then, the membranes were washed three times for 5 min with washing/dilution buffer (0.1% BSA in DPBS with 10 mM TCEP). While washing, it is important not to remove the entire volume so that the membrane will not dry out; therefore, 300 μl of wash buffer was added, and only 200 μl were removed. After the last washing step, the entire liquid was removed, after which 120 μl of GFP-PIF6-His_6_ (pHB111) with a concentration of 2.22 × 10^−7^ M in DPBS with 0.1% BSA and 10 mM TCEP was added immediately. Then, the samples were illuminated with custom-built LED panels, containing 660 nm (LED660N-03, Roithner Lasertechnik, Vienna, Austria; LH W5AM, Osram Opto Semiconductors, Regensburg, Germany) and 740 nm (LED740-01AU, Roithner Lasertechnik; LZ4-00R308, LED Engin, San Jose, CA) LEDs (*32*). The light intensities were measured using an AvaSpec-ULS2048 fiber-optic spectrometer (Avantes BV, Apeldoorn, Netherlands). A 660-nm illumination (15 μmol m^−2^ s^−1^) for 10 min followed by a washing step was done as previously described while applying 660-nm light. The membranes were kept in washing buffer until the assay was assembled. The analyte solution (pWW873) was prepared in DPBS with 0.1% BSA and 10 mM TCEP.

The sample holder for the nitrocellulose membranes were designed with SolidWorks 2021 (Dassault Systèmes SolidWorks Corp., France) and printed out of black acrylonitrile butadiene styrene with the Ultimaker 3 Extended (Ultimaker B.V., Netherlands). The assay was assembled as described in the following. First, each receiver membrane was put in the 3D-printed frames. Then, a sender membrane was added, and 40 μl of buffer or sample solution containing analyte was added immediately before assembly of the sender membrane. The frames were then added to a petri dish containing water to prevent evaporation during the assay time. The samples were then illuminated with either 740 nm (200 μmol m^−2^ s^−1^) or 660 nm (15 μmol m^−2^ s^−1^) for the indicated times. The nitrocellulose membranes were imaged using an ImageQuant LAS 4000 mini system (GE Healthcare, USA).

#### Biotin-binding proteins

Nitrocellulose membranes (Ø, 3 mm) were cut using a laser cutter as previously described and coated with 2 μg of anti-biotin antibody, nAv, or Strep-NC in DPBS and dried overnight at room temperature. The membranes were then blocked with either 5% (w/v) BSA for 30 min or 1.7% (w/v) casein for 5 min. The negative control samples (−), representing untreated membranes, were blocked with the respective blocking agent only. All samples were incubated with 120 μl of PhyB-biotin (pMH1105; 1 mg ml^−1^) with a final concentration of 0.5 mg ml^−1^ in DPBS with 10 mM TCEP for 30 min in the dark, followed by a GFP-PIF6-His_6_ (0.03 mg ml^−1^) in DPBS with 10 mM TCEP incubation during 660-nm illumination (15 μmol m^−2^ s^−1^) for 10 min with the previously described devices. Images were taken with an ImageQuant LAS 4000 mini system after 1 hour with 660 nm (15 μmol m^−2^ s^−1^) and 10 min with 740 nm (200 μmol m^−2^ s^−1^).

#### Homogeneous or spatially resolved release from nitrocellulose

Nitrocellulose membranes (Thermo Fisher Scientific, USA, 88018) were cut into squares (8 mm by 8 mm) and put into a 24-well plate. The samples were incubated in 300 μl of nAv (1.5 mg ml^−1^ in DPBS) for 30 min. After washing with DPBS for 2 min, the membranes were blocked with 300 μl of BSA solution (1% in DPBS) for 30 min. Then, 300 μl of biotinylated PhyB (pMH1105, 1 mg ml^−1^ in DPBS or Ni elution buffer) was added and incubated for 30 min in the dark. After washing (0.1% BSA in DPBS), 300 μl of GFP-PIF6 (pMH1450, 3 × 10^−2^ mg ml^−1^ in 5% BSA dissolved in DPBS) was incubated for 30 min under 660-nm illumination (46 μmol m^−2^ s^−1^; alternating 1 min on and 5 min off) from above. The samples were afterward washed during 660-nm illumination.

For spatially resolved release with a photomask, samples were put directly on the photomask, covered with buffer (0.1% BSA in DPBS), and illuminated from below with a 740-nm (77 μmol m^−2^ s^−1^) light for 2 min. Immediately after illumination, the samples were rinsed with DPBS and put into a new well containing DPBS. Experiments without a photo mask were conducted in a 24-well plate, where buffer with a volume of 300 μl was added before 740-nm (380 μmol m^−2^ s^−1^) or 660-nm (46 μmol m^−2^ s^−1^) illumination. Supernatant samples of 50 μl were taken at the indicated time points. Before visualization, DPBS was added to the samples during illumination of the respective wavelength and put into new well containing DPBS.

For testing the different PIF6 versions [GFP-PIF6 (pMH1450) and GFP-2xPIF6 (pMH1452)], the nitrocellulose membranes were prepared as described above. For the samples treated with biotin-GFP-PIF6, the PIF construct was added directly to nAv-functionalized and blocked membranes; this sample was only illuminated with red light. Fractions of the supernatant (50 μl) of all samples were taken at five time points during the 80-min illumination period. Fluorescence of the samples was measured using a Tecan Infinite M200 PRO (Tecan Group, Switzerland) plate reader (excitation wavelength, 488 nm, and emission wavelength, 530 nm); background fluorescence of the buffer (0.1% BSA in DPBS) was subtracted from each value before plotting.

#### Spatially resolved release from PMMA

PMMA (1.175 mm; Röhm GmbH, Germany, 99524 GT) rectangles (2 cm by 1.5 cm) were cut and put into a six-well plate. The samples were incubated with 1 ml of nAv (Thermo Fisher Scientific, USA, 31050) solution (1.5 mg ml^−1^ in DPBS) for 30 min. After washing with buffer (0.1% BSA in DPBS) for 2 min, PhyB (pMH1105, 2 mg ml^−1^ in Ni elution buffer) was incubated for 30 min in the dark. After washing, 1 ml of GFP-PIF6 (pMH1450, 1 mg ml^−1^ in Ni elution buffer) was incubated under 660-nm (46 μmol m^−2^ s^−1^; alternating 1 min on and 5 min off) illumination. The samples were afterward washed again. Samples were put directly on the photomask, covered with buffer (0.1% BSA in DPBS), and illuminated from below with 740 nm (77 μmol m^−2^ s^−1^) for 1 min. Immediately after illumination, the samples were rinsed with DPBS and put into a new well containing DPBS. For visualization, the GFP fluorescence of the PMMA slides was imaged using the ImageQuant LAS 4000 mini system.

#### Agarose beads

For 10 samples, 100 μl of nAv-functionalized agarose beads (Thermo Fisher Scientific, USA, 29202) was washed with buffer [0.1% (w/v) BSA in DPBS] by centrifugation at 500*g* for 2 min. Then, 800 μl of PhyB (pMH1105, 2 mg ml^−1^ in Ni elution buffer) was added and incubated for 30 min in the dark. PhyB solution was removed, and the samples were washed. mCh-PIF6 (600 μl; pMH23, 2.9 or 0.029 mg ml^−1^ in 0.1% BSA in DPBS) was added and incubated for 30 min under 660-nm illumination (46 μmol m^−2^ s^−1^; alternating 1 min on and 5 min off). After washing, the beads were resuspended in 2 ml of buffer (0.1% BSA in DPBS). For each sample, 200 μl of the resuspended beads was transferred into a 1.5-ml reaction tube.

The reaction tubes were illuminated for 1 hour at 660 nm (15 μmol m^−2^ s^−1^) or 740 nm (380 μmol m^−2^ s^−1^) from above. After illumination, the samples were spun down at 500*g* for 2 min, and 50 μl of the supernatant was taken for fluorescence measurements. mCh fluorescence (excitation wavelength, 587 nm, and emission wavelength, 625 nm) of the supernatant was detected using a microtiter plate reader Tecan Infinite M200 PRO (Tecan Group, Switzerland), and background fluorescence of the buffer (0.1% BSA in DPBS) was subtracted from each value before plotting.

#### Fibrin hydrogel

nAv was incorporated into a fibrin hydrogel and then loaded with biotinylated PhyB and PIF6-GFP during 660-nm illumination, as described in (*11*). The hydrogel was illuminated spatially resolved on a Zeiss LSM 880 laser scanning confocal microscope (10× objective EC Plan Neofluar NA 0.3) with a two-photon laser (Chameleon Discovery, Coherent) at 740 nm using the bleaching function of the Zen software. Global illumination was performed with a pE-4000 LED light source (CoolLED) at 660 nm (150 μmol s^−1^ m^−2^) or 740 nm (300 μmol s^−1^ m^−2^). PIF6-GFP was excited with a 488-nm laser, and emission was detected at 499 to 570 nm (pinhole 1 AU).

### PhotoBox with integrated electronics

The 3D-printed PhotoBox (*13*) served as an incubation unit by illumination with a 660-nm (12 μmol m^−2^ s^−1^) and 740-nm (185 μmol m^−2^ s^−1^) light and as imaging box with a smartphone-based readout. For the smartphone, a Samsung Galaxy A5 (2017) (Samsung Electronics, South Korea) was used. The images were taken during flashlight illumination with the integrated smartphone camera. The LEDs could be switched on and off via an Arduino Nano, which received the signal via an HC-05 Bluetooth module. The Arduino Nano is coded so that the user only needs to send either “660 nm” or “740 nm” via smartphone to the HC-05 to turn on/off the LEDs. Here, a self-programmed application for Android was used as a Bluetooth terminal to connect to HC-05.

The PhotoBox was designed with SolidWorks 2014 and printed out of black acrylonitrile butadiene styrene with the Ultimaker 3. The printed circuit board was designed with EAGLE software and produced by the company Beta LAYOUT Ltd. (Germany), and the soldering of the electronic components was done in-house.

### Data analysis

The images were analyzed using the software ImageJ (University of Wisconsin-Madison, USA). For the spatially resolved release experiments, the mean intensity values of nitrocellulose membranes/PMMA pieces for the illuminated and non-illuminated areas were analyzed with the area selection tool. To determine the final value of intensity, the mean background intensity of untreated nitrocellulose of PMMA was subtracted. For experiments with distinct nitrocellulose areas, the mean intensity values of the whole area were measured. The mean background intensity of untreated nitrocellulose was subtracted from each value.

## Supplementary Materials

### The PDF file includes:

Figs. S1 to S7 Tables S1 to S7 Legends for data files S1 and S2 Legends for movies S1 and S2

## References

[1] L. Syedmoradi, F. A. Gomez, Paper-based point-of-care testing in disease diagnostics. Bioanalysis 9, 841–843 (2017). 28644046 10.4155/bio-2017-0080

[2] X. Ding, S. F. Cheung, S. K. L. Cheng, D. T. Kamei, Paper-based systems for point-of-care biosensing. J. Lab. Autom. 20, 316–333 (2015). 25787805 10.1177/2211068215577197

[3] C. Dincer, R. Bruch, A. Kling, P. S. Dittrich, G. A. Urban, Multiplexed point-of-care testing–xPOCT. Trends Biotechnol. 35, 728–742 (2017). 28456344 10.1016/j.tibtech.2017.03.013 PMC5538621

[4] C. Dincer, R. Bruch, E. Costa-Rama, M. T. Fernández-Abedul, A. Merkoçi, A. Manz, G. A. Urban, F. Güder, Disposable sensors in diagnostics, food, and environmental monitoring. Adv. Mater. 31, 1806739 (2019). 10.1002/adma.201806739 31094032

[5] E. S. Burgie, R. D. Vierstra, Phytochromes: An atomic perspective on photoactivation and signaling. Plant Cell 26, 4568–4583 (2014). 25480369 10.1105/tpc.114.131623 PMC4311201

[6] T. Ziegler, A. Möglich, Photoreceptor engineering. Front. Mol. Biosci. 2, 30 (2015). 26137467 10.3389/fmolb.2015.00030 PMC4469895

[7] R. W. Smith, B. Helwig, A. H. Westphal, E. Pel, J. W. Borst, C. Fleck, Interactions between phyB and PIF proteins alter thermal reversion reactions in vitro. Photochem. Photobiol. 93, 1525–1531 (2017). 28503745 10.1111/php.12793

[8] D. Golonka, U. Gerken, J. Köhler, A. Möglich, The association kinetics encode the light dependence of arabidopsis phytochrome B interactions. J. Mol. Biol. 432, 4327–4340 (2020). 32534065 10.1016/j.jmb.2020.06.001

[9] S. Shimizu-Sato, E. Huq, J. M. Tepperman, P. H. Quail, A light-switchable gene promoter system. Nat. Biotechnol. 20, 1041–1044 (2002). 12219076 10.1038/nbt734

[10] A. Levskaya, O. D. Weiner, W. A. Lim, C. A. Voigt, Spatiotemporal control of cell signalling using a light-switchable protein interaction. Nature 461, 997–1001 (2009). 19749742 10.1038/nature08446 PMC2989900

[11] H. M. Beyer, O. S. Thomas, N. Riegel, M. D. Zurbriggen, W. Weber, M. Hörner, Generic and reversible opto-trapping of biomolecules. Acta Biomater. 79, 276–282 (2018). 30165200 10.1016/j.actbio.2018.08.032

[12] M. Hörner, J. Becker, R. Bohnert, M. Baños, C. Jerez-Longres, V. Mühlhäuser, D. Härrer, T. W. Wong, M. Meier, W. Weber, A photoreceptor-based hydrogel with red light-responsive reversible sol-gel transition as transient cellular matrix. Adv. Mater. Technol. 8, 2300195 (2023).

[13] F. Wieland, R. Bruch, M. Bergmann, S. Partel, G. A. Urban, C. Dincer, Enhanced protein immobilization on polymers—A plasma surface activation study. Polymers 12, 104 (2020). 31947987 10.3390/polym12010104 PMC7023393

[14] M. Jung Kim, I. Haizan, M. J. Ahn, D.-H. Park, J.-H. Choi, Recent advances in lateral flow assays for viral protein detection with nanomaterial-based optical sensors. Biosensors 14, 197 (2024). 38667190 10.3390/bios14040197 PMC11048458

[15] N. Abu, N. M. Bakhori, R. H. Shueb, Lateral flow assay for hepatitis B detection: A review of current and new assays. Micromachines 14, 1239 (2023). 37374824 10.3390/mi14061239 PMC10301844

[16] C. A. Holstein, A. Chevalier, S. Bennett, C. E. Anderson, K. Keniston, C. Olsen, B. Li, B. Bales, D. R. Moore, E. Fu, D. Baker, P. Yager, Immobilizing affinity proteins to nitrocellulose: A toolbox for paper-based assay developers. Anal. Bioanal. Chem. 408, 1335–1346 (2016). 26427504 10.1007/s00216-015-9052-0

[17] X. Zhuang, Z. Zhao, X. Feng, G. C. Y. Lui, D. Chan, S. Lee, I.-M. Hsing, Integrating magnetic-bead-based sample extraction and molecular barcoding for the one-step pooled RT-qPCR assay of viral pathogens without retesting. Anal. Chem. 95, 6182–6190 (2023). 37005794 10.1021/acs.analchem.3c00885 PMC10100384

[18] D. Stern, T. C. Meyer, F. Treindl, H. W. Mages, M. Krüger, M. Skiba, J. P. Krüger, C. M. Zobel, M. Schreiner, M. Grossegesse, T. Rinner, C. Peine, A. Stoliaroff-Pépin, T. Harder, N. Hofmann, J. Michel, A. Nitsche, S. Stahlberg, A. Kneuer, A. Sandoni, U. Kubisch, M. Schlaud, A. Mankertz, T. Schwarz, V. M. Corman, M. A. Müller, C. Drosten, K. De La Rosa, L. Schaade, M. B. Dorner, B. G. Dorner, A bead-based multiplex assay covering all coronaviruses pathogenic for humans for sensitive and specific surveillance of SARS-CoV-2 humoral immunity. Sci. Rep. 13, 21846 (2023). 38071261 10.1038/s41598-023-48581-9 PMC10710470

[19] P. G. Gottschalk, J. R. Dunn, The five-parameter logistic: A characterization and comparison with the four-parameter logistic. Anal. Biochem. 343, 54–65 (2005). 15953581 10.1016/j.ab.2005.04.035

[20] J. W. A. Findlay, R. F. Dillard, Appropriate calibration curve fitting in ligand binding assays. AAPS J. 9, E260–E267 (2007). 17907767 10.1208/aapsj0902029 PMC2751416

[21] J. Zheng, S. Q. Zhao, X. T. Xu, K. Zhang, Detection of bisphenol A in water samples using ELISA determination method. Water Sci. Technol. 11, 55–60 (2011).

[22] R. Galarini, F. Diana, S. Moretti, B. Puppini, G. Saluti, L. Persic, Development and validation of a new qualitative ELISA screening for multiresidue detection of sulfonamides in food and feed. Food Control 35, 300–310 (2014).

[23] L. Han, Y.-T. Li, J.-Q. Jiang, R.-F. Li, G.-Y. Fan, J.-M. Lv, Y. Zhou, W.-J. Zhang, Z.-L. Wang, Development of a direct competitive ELISA kit for detecting deoxynivalenol contamination in wheat. Molecules 25, 50 (2020). 10.3390/molecules25010050 PMC6983206 31877851

[24] S. M. Moosavi, S. Ghassabian, “Linearity of calibration curves for analytical methods: A review of criteria for assessment of method reliability, *in Calibration and Validation of Analytical Methods—A Sampling of Current Approaches* (InTech, 2018); www.intechopen.com/books/calibration-and-validation-of-analytical-methods-a-sampling-of-current-approaches/linearity-of-calibration-curves-for-analytical-methods-a-review-of-criteria-for-assessment-of-method.

[25] J. R. Horsley, J. Yu, K. L. Wegener, C. Hoppmann, K. Rück-Braun, A. D. Abell, Photoswitchable peptide-based ‘on-off’ biosensor for electrochemical detection and control of protein-protein interactions. Biosens. Bioelectron. 118, 188–194 (2018). 30077871 10.1016/j.bios.2018.07.057

[26] B. Adelizzi, V. Gielen, T. Le Saux, P. Dedecker, L. Jullien, Quantitative model for reversibly photoswitchable sensors. ACS Sens. 6, 1157–1165 (2021). 33565309 10.1021/acssensors.0c02414 PMC8008439

[27] B. Nilsson, T. Moks, B. Jansson, L. Abrahmsén, A. Elmblad, E. Holmgren, C. Henrichson, T. A. Jones, M. Uhlén, A synthetic IgG-binding domain based on staphylococcal protein a. Protein Eng. Des. Sel. 1, 107–113 (1987). 10.1093/protein/1.2.107 3507693

[28] M. Hörner, C. Jerez-Longres, A. Hudek, S. Hook, O. S. Yousefi, W. W. A. Schamel, C. Hörner, M. D. Zurbriggen, H. Ye, H. J. Wagner, W. Weber, Spatiotemporally confined red light-controlled gene delivery at single-cell resolution using adeno-associated viral vectors. Sci. Adv. 7, eabf0797 (2021). 34134986 10.1126/sciadv.abf0797 PMC8208708

[29] G. Guntas, R. A. Hallett, S. P. Zimmerman, T. Williams, H. Yumerefendi, J. E. Bear, B. Kuhlman, Engineering an improved light-induced dimer (iLID) for controlling the localization and activity of signaling proteins. Proc. Natl. Acad. Sci. U.S.A. 112, 112–117 (2015). 25535392 10.1073/pnas.1417910112 PMC4291625

[30] D. G. Gibson, L. Young, R.-Y. Chuang, J. C. Venter, C. A. Hutchison, H. O. Smith, Enzymatic assembly of DNA molecules up to several hundred kilobases. Nat. Methods 6, 343–345 (2009). 19363495 10.1038/nmeth.1318

[31] M. Hörner, K. Gerhardt, P. Salavei, P. Hoess, D. Härrer, J. Kaiser, J. J. Tabor, W. Weber, Production of phytochromes by high-cell-density *E. coli* fermentation. ACS Synth. Biol., 8, 2442–2450 (2019). 31526004 10.1021/acssynbio.9b00267

[32] K. Müller, M. D. Zurbriggen, W. Weber, Control of gene expression using a red- and far-red light-responsive bi-stable toggle switch. Nat. Protoc. 9, 622–632 (2014). 24556785 10.1038/nprot.2014.038

