# Supplementary material for: OptoAssay—Light-controlled dynamic bioassay using optogenetic switches
Source: Sci Adv. 2024 Sep 25;10(39):eadp0911. doi: 10.1126/sciadv.adp0911 (PMC11423887; doi:10.1126/sciadv.adp0911)
Supplement: Supplementary file 1 — Figs. S1 to S7 Tables S1 to S7 Legends for data files S1 and S2 Legends for movies S1 and S2 [file sciadv.adp0911_sm.pdf]

Science Advances
AAAS

Supplementary Materials for

OptoAssay—Light-controlled dynamic bioassay using optogenetic switches

Nadine Urban *et al.*

Corresponding author: Wilfried Weber, wilfried.weber@leibniz-inm.de; Can Dincer, dincer@imtek.de

Sci. Adv. **10**, eadp0911 (2024)
DOI: 10.1126/sciadv.adp0911

The PDF file includes:

Figs. S1 to S7
Tables S1 to S7
Legends for data files S1 and S2
Legends for movies S1 and S2

Other Supplementary Material for this manuscript includes the following:

Data files S1 and S2
Movies S1 and S2

Experimental Figures

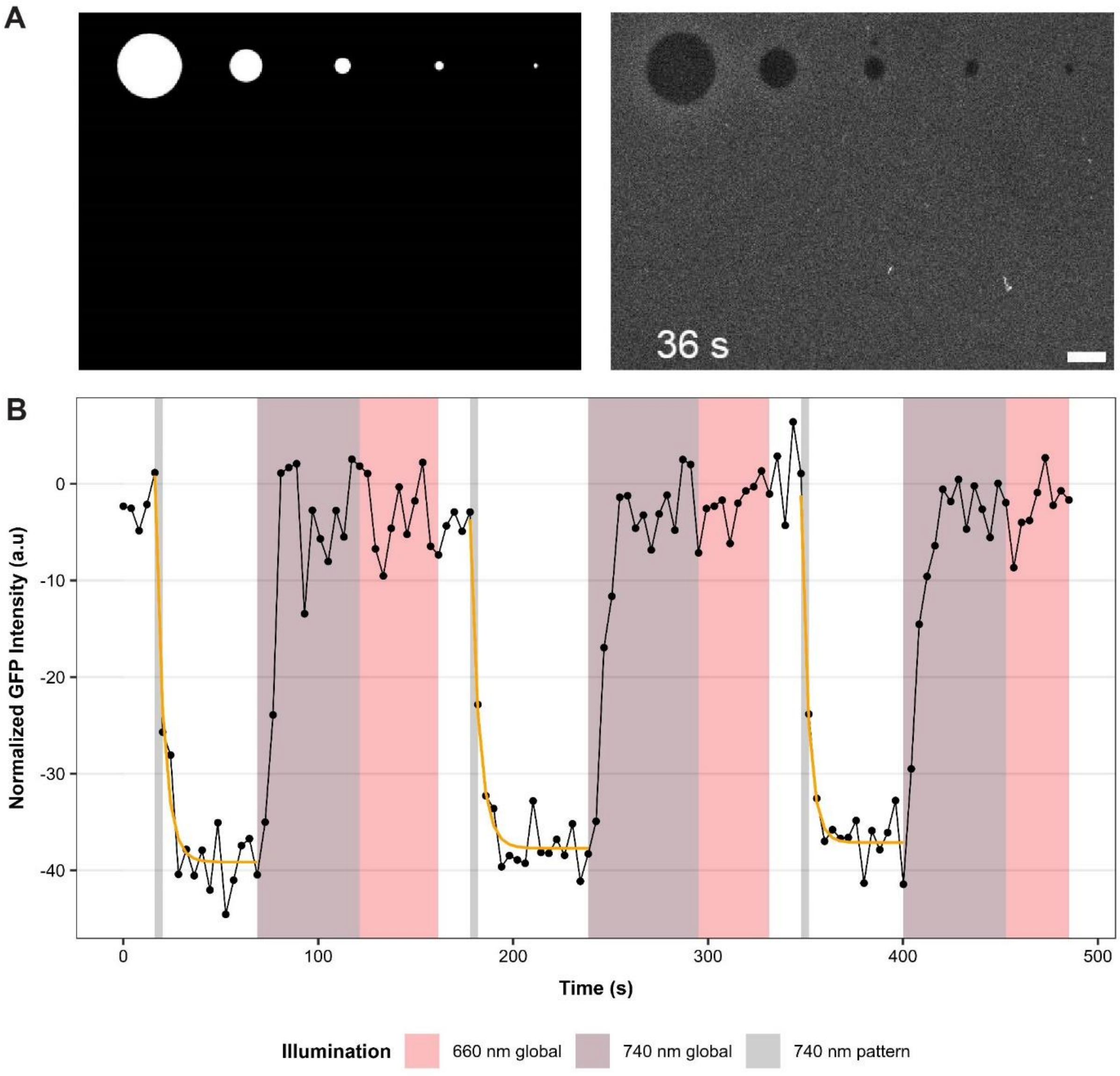

Figure S1. PhyB/PIF6 kinetics in a hydrogel. A) Illumination pattern (left image) indicating the with 740 nm illuminated areas (white) of a fibrin hydrogel that was loaded with PhyB and GFP-PIF6 (GFP fluorescence is shown on the right side). Scale bar, 50 µm. B) Normalized GFP intensity of the second from the right circular area (diameter 12 µm, due to the small diameter diffusion can be neglected) over time. The hydrogel was either illuminated locally (see illumination pattern) with 740 nm or globally with 740 or 660 nm light. A video of all images of the time series can be found in the supplementary materials (Video S2). The orange lines show fits to the normalized GFP intensity after localized 740 nm illumination using an exponential decay function. The fitted curves were used to determine the mean half-life of the GFP-PIF6 release as $\tau_{1/2}$ = 2.91 ± 0.27 s.

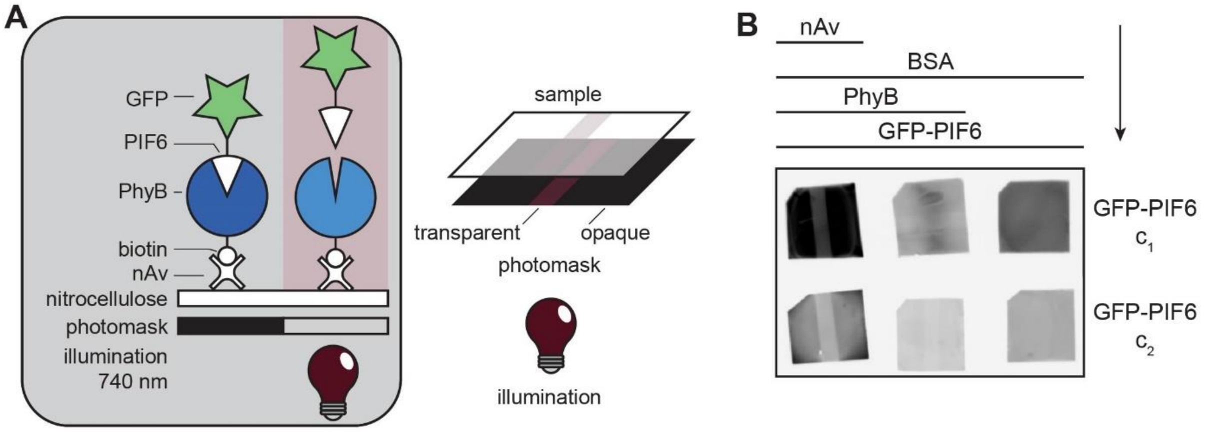

Figure S2. Functionality of the PhyB/PIF6 System on a Nitrocelulose Substrate. A) Experimental setup. The nitrocellulose membrane was incubated with nAv, then blocked with BSA. After addition of PhyB, GFP-PIF6 was immobilized on PhyB during red light (660 nm) illumination and finally released through far-red light (740 nm) illumination. The scheme shows how the sample is luminated trough the transparent part of the photomask. **B)** GFP fluorescence of nitrocellulose membranes after the addition of GFP-PIF6 and illumination with far-red light (740 nm) through a photomask for 2 min. The samples were either treated with nAv or left untreated before PhyB immobilisation. Then, the samples were incubated with two different concentrations of GFP-PIF6 ($c_1 = 3 \times 10^{-2}$ mg mL^{-1} and $c_2 = 0.6 \times 10^{-2}$ mg mL^{-1}) during red light illumination. A control for unspecific binding of GFP-PIF, comprising a blocked membrane plus GFP-PIF, was prepared as well. The samples were covered with buffer before illumination with far-red light through a photomask.

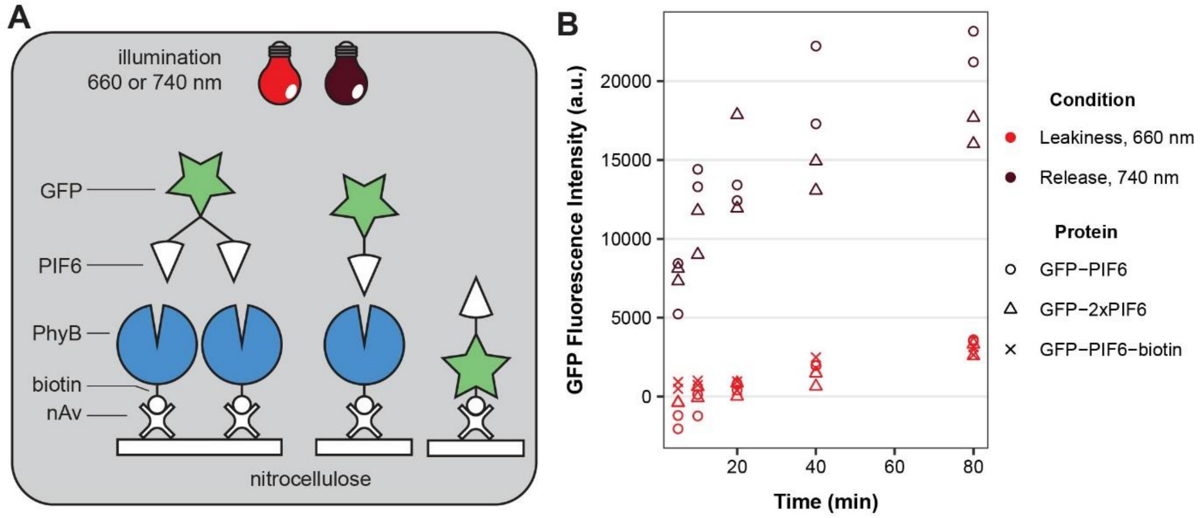

Figure S3. Different PIF6 Versions. A) Experimental setup. Neutravidin (nAv) treated nitrocellulose membranes were either incubated with PhyB before addition of GFP-PIF6 and GFP-2xPIF6, respectively, or GFP-PIF6-biotin was directly immobilized on the nAv-coated membranes. The samples were then illuminated with red (660 nm) or far-red (740 nm). **B)** Release kinetics of the model cargo GFP from functionalized nitrocellulose. The samples containing different cargo proteins GFP-PIF6, GFP-PIF6-biotin or GFP-2xPIF6 (3×10^{-2} mg mL^{-1}) were illuminated from above in a 24-well plate, covered with buffer, for 80 min either with red (5 µmol m^{-2} s^{-1}) or far-red light (380 µmol m^{-2} s^{-1}). Graph shows the GFP fluorescence of supernatant over a time range of 80 min in a.u. 50 µL of the supernatant at time points 5, 10, 40 and 80 min were taken for GFP fluorescence measurements. The background fluorescence of the buffer was subtracted from each value. Red markings represent samples illuminated with red light, while dark-red ones the samples illuminated with far-red light.

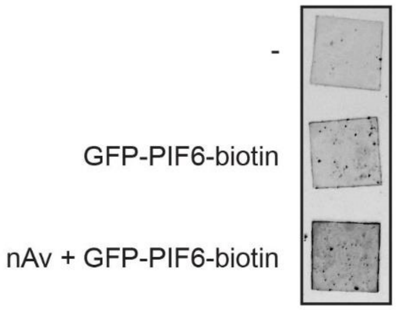

Figure S4. Adsorption Test on PMMA Samples. GFP fluorescence images of PMMA samples. The first sample was only treated with PBS (-) while the second sample was treated with biotinylated GFP-PIF6 only. On the third sample, nAv was first adsorbed followed by blocking with 5% BSA. Then, GFP-PIF6-biotin was added. The images were taken after washing the samples.

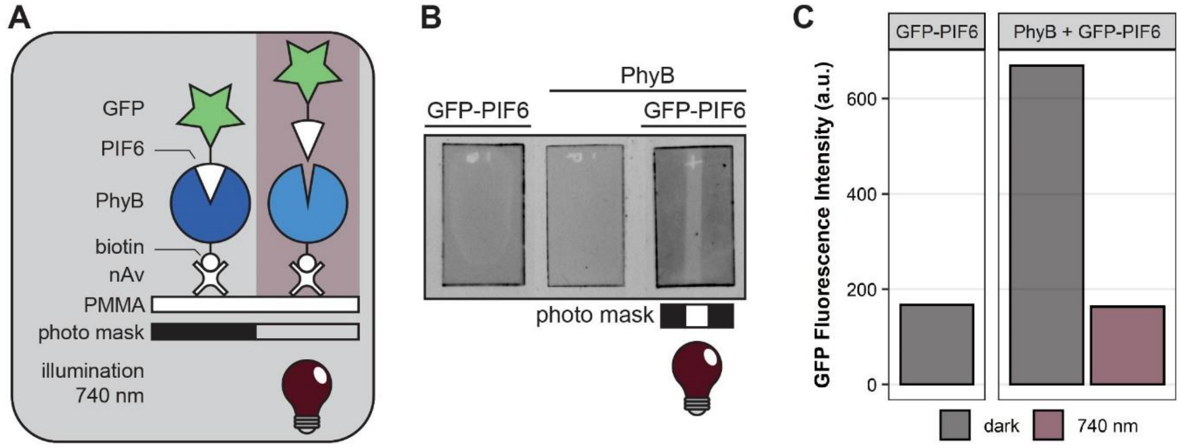

Figure S5. Release Experiments on PMMA Substrate. A) Experimental setup of the spatially resolved release of the competitor complex PIF6-GFP from PMMA. Competitor complex was immobilized via biotinylated PhyB on PMMA treated with neutravidin (nAv). The samples were illuminated from below with far-red light (740 nm) through a photomask. **B)** GFP fluorescence images of PMMA samples. The first sample was only treated with GFP-PIF6 and the middle sample was only treated with biotinylated PhyB after nAv treatment and blocking. For the right most sample, first nAv was adsorbed on PMMA, then biotinylated PhyB was added to the. After blocking, GFP-PIF6 was incubated during red light (660 nm) illumination. This sample was then put on a photomask and after addition of buffer to cover the whole area, the sample was illuminated through photomask with far-red light from below for

1 min. The other samples were left in the dark. **C)** Quantification of fluorescence intensity of the PMMA substrate measured from B in a.u. The background fluorescence of the PhyB only sample was subtracted from all samples before plotting. Black bars display the intensity of the not-illuminated samples/areas; the dark-red bar displays the intensity of the illuminated area.

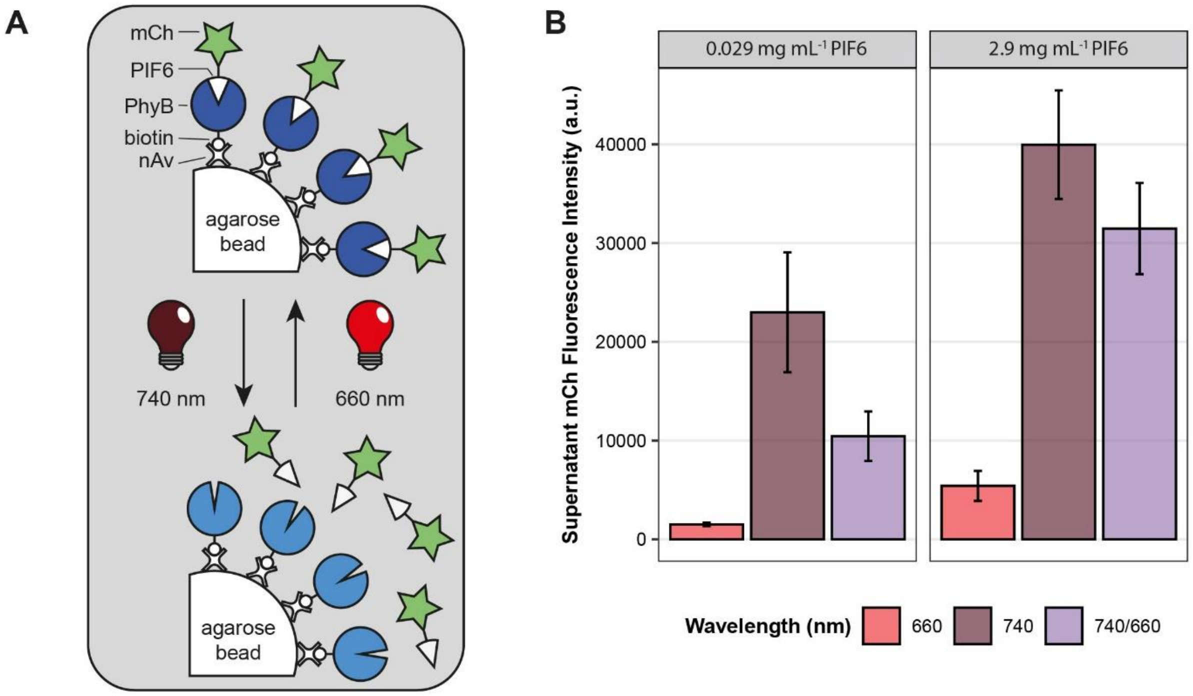

Figure S6. Release and Rebinding Experiments on Agarose Beads. A) Experimental setup of release and rebinding of the competitor complex PIF6-mCh from and to PhyB-functionalized agarose beads. Illumination with far-red light (740 nm) leads to a release of competitor from PhyB that is coupled to the beads via neutravidin-biotin interaction; during red light (660 nm) illumination mCh-PIF6 and PhyB are associated. **B)** For the preparation, 10 µl PhyB coated beads were incubated with two different concentrations of mCh-PIF6 (0.029 or 2.9 mg ml^{-1}) under red light illumination. For this experiment, the loaded beads were incubated in buffer and either illuminated with red or far-red light for 1 h or first with far-red light for 30 min and then with red light for 30 min. The mCh fluorescence intensity was measured in a.u. from the supernatant of the sedimented beads. The background fluorescence of the buffer was subtracted from each value. The bars represent the mean of n = 3 samples, error bars represent the standard deviation.

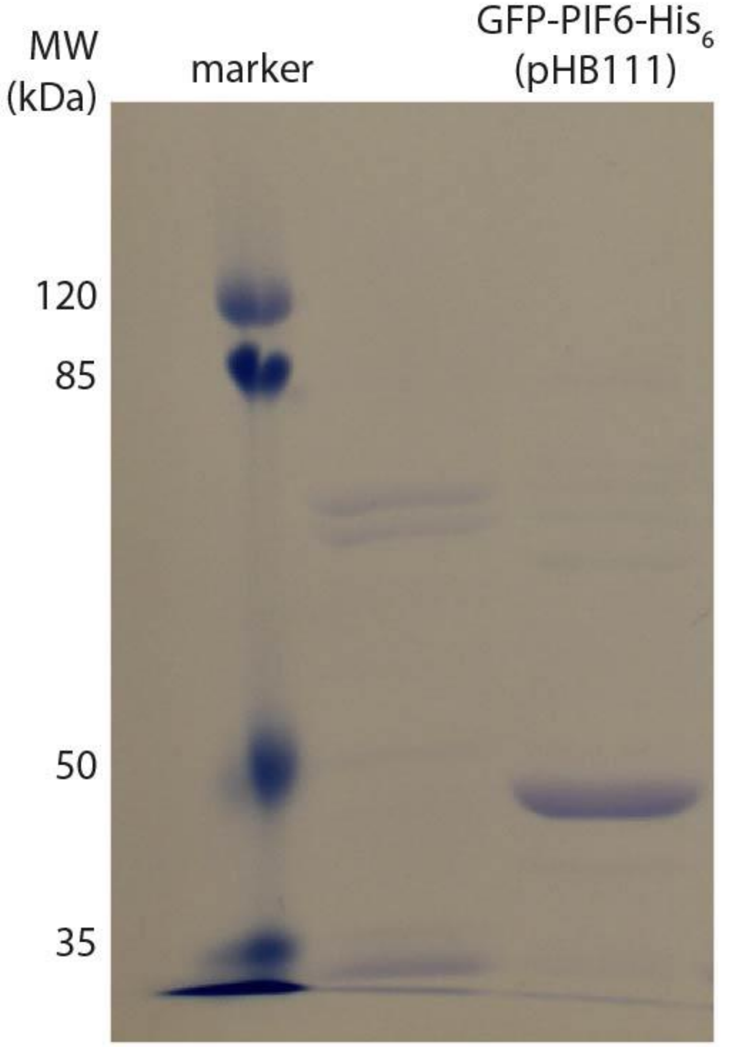

Figure S7. SDS PAGE of GFP-PIF6-His$_6$. After purification, the protein was mixed with 5x loading dye and loaded on a 9% SDS gel and run for 1 h at 120 V. A Coomassie staining was subsequently carried out. The calculated molecular weight of GFP-PIF6-His$_6$ (pHB111) is 40 kDa.

Experimental Data

Measurement data from **Figure 3A** and **Figure S1** can be found in separate files (Data_Fig3A.xlsx, Data_FigS1.xlsx).

Table S1. Measurement data from Figure 2A, B. GFP Fluorescence Intensity I (a.u).

Protein	Image	Blocking agent	I (a.u)
Anti-biotin	Immediately	BSA	2002.4
Anti-biotin	Immediately	BSA	2123.4
nAv	Immediately	BSA	1922.3
nAv	Immediately	BSA	2120.1
Strep-NC	Immediately	BSA	1958.9
Strep-NC	Immediately	BSA	1925
-	Immediately	BSA	1637.5
-	Immediately	BSA	1827.5
Background	Immediately	BSA	1179.1
Background	Immediately	BSA	1169.4
Anti-biotin	660 nm	BSA	1908.6
Anti-biotin	660 nm	BSA	2030.4
nAv	660 nm	BSA	1880.6
nAv	660 nm	BSA	2092.4
Strep-NC	660 nm	BSA	1989.6
Strep-NC	660 nm	BSA	1915.4
-	660 nm	BSA	1629
-	660 nm	BSA	1841.1
Background	660 nm	BSA	1186.8
Background	660 nm	BSA	1170.4
Anti-biotin	740 nm	BSA	1792.9
Anti-biotin	740 nm	BSA	1877.5
nAv	740 nm	BSA	1742.6
nAv	740 nm	BSA	1814.5
Strep-NC	740 nm	BSA	1768.6
Strep-NC	740 nm	BSA	1742.3
-	740 nm	BSA	1621.7
-	740 nm	BSA	1806
Background	740 nm	BSA	1180.5
Background	740 nm	BSA	1168.1
Anti-biotin	Washing	BSA	1789.7
Anti-biotin	Washing	BSA	1880.2
nAv	Washing	BSA	1753.6
nAv	Washing	BSA	1765.2
Strep-NC	Washing	BSA	1741.1
Strep-NC	Washing	BSA	1713.1
-	Washing	BSA	1631

-	Washing	BSA	1807.8
Background	Washing	BSA	1178.8
Background	Washing	BSA	1172.1
Anti-biotin	Immediately	Casein	1454.4
Anti-biotin	Immediately	Casein	1430.5
nAv	Immediately	Casein	1295.5
nAv	Immediately	Casein	1303.1
Strep-NC	Immediately	Casein	1864.5
Strep-NC	Immediately	Casein	1674.3
-	Immediately	Casein	1203.1
-	Immediately	Casein	1186.3
Background	Immediately	Casein	1194.7
Background	Immediately	Casein	1175
Anti-biotin	660 nm	Casein	1415.8
Anti-biotin	660 nm	Casein	1394.7
nAv	660 nm	Casein	1255.6
nAv	660 nm	Casein	1270.8
Strep-NC	660 nm	Casein	1791.5
Strep-NC	660 nm	Casein	1564.4
-	660 nm	Casein	1197.6
-	660 nm	Casein	1175.8
Background	660 nm	Casein	1188.9
Background	660 nm	Casein	1173.8
Anti-biotin	740 nm	Casein	1329.5
Anti-biotin	740 nm	Casein	1333.9
nAv	740 nm	Casein	1218.3
nAv	740 nm	Casein	1217.3
Strep-NC	740 nm	Casein	1450.9
Strep-NC	740 nm	Casein	1372.4
-	740 nm	Casein	1193
-	740 nm	Casein	1170.5
Background	740 nm	Casein	1183.8
Background	740 nm	Casein	1175.6
Anti-biotin	Washing	Casein	1327.7
Anti-biotin	Washing	Casein	1317.7
nAv	Washing	Casein	1196.1
nAv	Washing	Casein	1197.2
Strep-NC	Washing	Casein	1370
Strep-NC	Washing	Casein	1329.5
-	Washing	Casein	1194.7
-	Washing	Casein	1175.2
Background	Washing	Casein	1189.5
Background	Washing	Casein	1172.5

Table S2. Measurement data from Figure 2C and D. GFP Fluorescence Intensity I (a.u).

Image	Condition	Membrane	I (a.u)
Initial state	Control	Sender	1920.1
Initial state	Control	Sender	1789.6
Initial state	Rebinding	Sender	1645.5
Initial state	Rebinding	Sender	1583.4
660 nm	Control	Sender	1916.2
660 nm	Control	Sender	1788.9
660 nm	Rebinding	Sender	1885.9
660 nm	Rebinding	Sender	1760.2
Initial state	Control	Receiver	1362.8
Initial state	Control	Receiver	1351.9
Initial state	Rebinding	Receiver	1461.8
Initial state	Rebinding	Receiver	1556.6
660 nm	Control	Receiver	1378.3
660 nm	Control	Receiver	1359.1
660 nm	Rebinding	Receiver	1485.7
660 nm	Rebinding	Receiver	1625.2
Background	Background	Background	1262.8

Table S3: Measured experimental data from Figure 3B. GFP Fluorescence Intensity I (a.u).

Image	Analyte concentration (pg ml^{-1})	I (a.u)
30 min release	6.81e+06	1630.9
30 min release	6.81e+06	1545.1
30 min release	6.81e+06	1615.5
Rebinding	6.81e+06	1619.3
Rebinding	6.81e+06	1505.6
Rebinding	6.81e+06	1559
30 min release	1.36e+05	1511.1
30 min release	1.36e+05	1640.7
30 min release	1.36e+05	1671.4
Rebinding	1.36e+05	1517.3
Rebinding	1.36e+05	1607.2
Rebinding	1.36e+05	1708.6
30 min release	1.09e+03	1600.5
30 min release	1.09e+03	1532.4
30 min release	1.09e+03	1553
Rebinding	1.09e+03	1601.7
Rebinding	1.09e+03	1514.8
Rebinding	1.09e+03	1562.9
30 min release	5.45e+01	1801.4
30 min release	5.45e+01	1854.7
30 min release	5.45e+01	1738.6

Rebinding	5.45e+01	1831.8
Rebinding	5.45e+01	1918.6
Rebinding	5.45e+01	1811.1
Background	0.00e+00	1262.8

Table S4. Exponential decay fitting parameters and calculated half-life $T_{1/2}$ from Figure S1.

Decay Phase	$f(x)_0$	P	K	t	$T_{1/2}$
1	0.859	-39.14	0.2331	16.171	2.973604
2	-2.753	-37.71	0.2206	177.76	3.1421
3	1.118	-37.12	0.266	347.44	2.605816

Table S5. Measurement data from Figure S3. GFP Fluorescence Intensity I (a.u).

Illumination Wavelength	Protein	Time (min)	I (a.u)
660 nm	GFP-PIF6	5	3762
660 nm	GFP-PIF6	5	4608
660 nm	GFP-PIF6-linker-PIF6	5	5434
660 nm	GFP-PIF6-linker-PIF6	5	5415
660 nm	GFP-PIF6-biotin	5	6303
660 nm	GFP-PIF6-biotin	5	6740
740 nm	GFP-PIF6	5	11040
740 nm	GFP-PIF6	5	14250
740 nm	GFP-PIF6-linker-PIF6	5	13931
740 nm	GFP-PIF6-linker-PIF6	5	13132
660 nm	GFP-PIF6	10	5939
660 nm	GFP-PIF6	10	4577
660 nm	GFP-PIF6-linker-PIF6	10	5723
660 nm	GFP-PIF6-linker-PIF6	10	6424
660 nm	GFP-PIF6-biotin	10	6542
660 nm	GFP-PIF6-biotin	10	6816
740 nm	GFP-PIF6	10	19115
740 nm	GFP-PIF6	10	20226
740 nm	GFP-PIF6-linker-PIF6	10	14816
740 nm	GFP-PIF6-linker-PIF6	10	17597
660 nm	GFP-PIF6	20	6218
660 nm	GFP-PIF6	20	6633
660 nm	GFP-PIF6-linker-PIF6	20	5824
660 nm	GFP-PIF6-linker-PIF6	20	6654
660 nm	GFP-PIF6-biotin	20	6194
660 nm	GFP-PIF6-biotin	20	6764
740 nm	GFP-PIF6	20	19232
740 nm	GFP-PIF6	20	18237
740 nm	GFP-PIF6-linker-PIF6	20	17747

740 nm	GFP-PIF6-linker-PIF6	20	23681
660 nm	GFP-PIF6	40	7765
660 nm	GFP-PIF6	40	7770
660 nm	GFP-PIF6-linker-PIF6	40	6457
660 nm	GFP-PIF6-linker-PIF6	40	7284
660 nm	GFP-PIF6-biotin	40	8292
660 nm	GFP-PIF6-biotin	40	7594
740 nm	GFP-PIF6	40	23109
740 nm	GFP-PIF6	40	28041
740 nm	GFP-PIF6-linker-PIF6	40	18889
740 nm	GFP-PIF6-linker-PIF6	40	20745
660 nm	GFP-PIF6	80	9312
660 nm	GFP-PIF6	80	9415
660 nm	GFP-PIF6-linker-PIF6	80	9125
660 nm	GFP-PIF6-linker-PIF6	80	8387
660 nm	GFP-PIF6-biotin	80	8451
660 nm	GFP-PIF6-biotin	80	8949
740 nm	GFP-PIF6	80	28981
740 nm	GFP-PIF6	80	27029
740 nm	GFP-PIF6-linker-PIF6	80	21835
740 nm	GFP-PIF6-linker-PIF6	80	23501
	Backgound		5828
	Backgound		5411
	Backgound		6192

Table S6. Measurement data from Figure S6. mCh Fluorescence Intensity I (a.u).

PIF6- mCh concentration (mg mL^{-1})	Illlumination wavelength (nm)	I (a.u)
2.9	660	3675
2.9	660	6228
2.9	660	6374
2.9	740	33700
2.9	740	42296
2.9	740	43943
2.9	740/660	26164
2.9	740/660	33973
2.9	740/660	34343
-	Background	12
-	Background	15
-	Background	17
0.029	660	1675
0.029	660	1574
0.029	660	1332
0.029	740	29892

0.029	740	18443
0.029	740	20696
0.029	740/660	7987
0.029	740/660	10393
0.029	740/660	12990

Data Fitting

OptoAssay

An immunoassay serves the purpose of quantifying the concentration of a specific substance in a given sample. This is accomplished by comparing the response obtained from the assay for an unknown concentration of the substance to a calibration curve, commonly known as the "standard curve" (*19*).

The response of an immunoassay typically follows an "S-shaped" or sigmoidal relationship with the concentration (*19*)'(*20*). Various models can be employed to fit this type of data, including mechanistic models based on the law of mass action, or empirical models that utilize mathematical functions to approximate the distribution of the response data (*33*).Among these models, the four-parameter and five-parameter logistic curve models (4PL and 5PL, respectively) are commonly used to represent immunoassay data. These models employ regression analysis to minimize the disparity between the modelled and observed responses (*19*).

The equation describing the 4PL model, which was used in our case, is as follows:

Equation 1

$$Y = A_2 + \frac{(A_1 - A_2)}{1 + \left(\frac{X}{x_0}\right)^p}$$

where Y represents the obtained response, A_1 denotes the response at zero-analyte concentration, A_2 signifies the response at infinite analyte concentration, X stands for the analyte concentration, x_0 represents the inflection point (IC50), and p is the slope factor (*20*).

The limit of detection (LOD) or lowest limit of detection (LLOD), representing the sensitivity of a competitive assay is determined by the calculated curve and the standard deviation (SD) of the signal response (Y), the zero-analyte concentration (blank) and the lowest concentrated sample (LCS). The LOD for a competitive assay format is calculated as followed:

Equation 2

$$Y_{LOD} = Y(Mean_{blank}) - 1.645 * Y(SD_{blank}) - 1.645 * Y(SD_{LCS})$$

Equation 3

$$LOD = X_{LOD} = x_0 \left(\left(\frac{Y_{LOD} - A_{max}}{A_{min} - Y_{LOD}} \right)^{1/p} \right)$$

The precision of measurements is represented by the coefficient of variation (CV):

Equation 4

$$CV = \frac{Standard\ deviation\ (Measurement\ points)}{Average\ value\ (Measurement\ points)}$$

PhyB/PIF6 kinetics in a hydrogel

An exponential decay function

Equation 5

$$f(x) = (f(x)_0 - P)e^{-K(x-t)} + P$$

where $f(x)_0$ represents the initial value of $f(x)$ at the starting time t, P is the asymptotic value that $f(x)$ approaches with increasing time x and K is the decay constant, was fitted from start of the 740 nm mask illumination to the start of 740 nm global illumination using the Levenberg- Marquardt

Nonlinear Least-Squares algorithm (*34*). The decay constant K was subsequently used to calculate the half-life time $\tau_{1/2}$.

Equation 4

$$\tau_{1/2} = \frac{\ln(2)}{K}$$

This was done for all of the three decay phases. Then, the mean and standard deviation from the $\tau_{1/2}$ values was calculated.

Plasmids

Table S7. Nucleic acid sequence of plasmids used in this study.

Plasmid	Nucleic acid sequence 5' →3'
pMH023<br><br>pRSET backbone<br><br>zz-linker<br><br>mCherry<br><br>PIF6(1-100)<br><br>6xHis-tag	…GGAGATATACATATGGCGCAACACGATGAAGCCGTAGACA<br>ACAAATTCAACAAAGAACAACAAAACGCGTTCTATGAGATC<br>TTACATTTACCTAACTTAAACGAAGAACAACGAAACGCCTT<br>CATCCAAAGTTTAAAAGATGACCCAAGCCAAAGCGCTAACC<br>TTTTAGCAGAAGCTAAAAAGCTAAATGATGCTCAGGCGCCG<br>AAAGTAGACAACAAATTCAACAAAGAACAACAAAACGCGTT<br>CTATGAGATCTTACATTTACCTAACTTAAACGAAGAACAAC<br>GAAACGCCTTCATCCAAAGTTTAAAAGATGACCCAAGCCAA<br>AGCGCTAACCTTTTAGCAGAAGCTAAAAAGCTAAATGATGC<br>TCAGGCGCCGAAAGTAGACGCGAATTCGAGCGGTGCAGGTA<br>GCGGTTCTGGCATGGTGAGCAAGGGCGAGGAGGATAACATG<br>GCCATCATCAAGGAGTTCATGCGCTTCAAGGTGCACATGGA

	GGGCTCCGTGAACGGCCACGAGTTCGAGATCGAGGGCGAGG<br>GCGAGGGCCGCCCCTACGAGGGCACCCAGACCGCCAAGCTG<br>AAGGTGACCAAGGGTGGCCCCCTGCCCTTCGCCTGGGACAT<br>CCTGTCCCCTCAGTTCATGTACGGCTCCAAGGCCTACGTGA<br>AGCACCCCGCCGACATCCCCGACTACTTGAAGCTGTCCTTC<br>CCCGAGGGCTTCAAGTGGGAGCGCGTGATGAACTTCGAGGA<br>CGGCGGCGTGGTGACCGTGACCCAGGACTCCTCCCTGCAGG<br>ACGGCGAGTTCATCTACAAGGTGAAGCTGCGCGGCACCAAC<br>TTCCCCTCCGACGGCCCCGTAATGCAGAAGAAGACCATGGG<br>CTGGGAGGCCTCCTCCGAGCGGATGTACCCCGAGGACGGCG<br>CCCTGAAGGGCGAGATCAAGCAGAGGCTGAAGCTGAAGGAC<br>GGCGGCCACTACGACGCTGAGGTCAAGACCACCTACAAGGC<br>CAAGAAGCCCGTGCAGCTGCCCGGCGCCTACAACGTCAACA<br>TCAAGTTGGACATCACCTCCCACAACGAGGACTACACCATC<br>GTGGAACAGTACGAACGCGCCGAGGGCCGCCACTCCACCGG<br>CGGCATGGACGAGCTGTACAAGGGCTCCGCAGGTTCTGCTG<br>GTATGATGTTCTTACCAACCGATTATTGTTGCAGGTTAAGC<br>GATCAAGAGTATATGGAGCTTGTGTTTGAGAATGGCCAGAT<br>TCTTGCAAAGGGCCAAAGATCCAACGTTTCTCTGCATAATC<br>AACGTACCAAATCGATCATGGATTTGTATGAGGCAGAGTAT<br>AACGAGGATTTCATGAAGAGTATCATCCATGGTGGTGGTGG<br>TGCCATCACAAATCTCGGGGACACGCAGGTTGTTCCACAAA<br>GTCATGTTGCTGCTGCCCATGAAACAAACATGTTGGAAAGC

	AATAAACATGTTGACCATCATCACCATCACCATTAAAAGCT<br>TG…
pMH1409<br>pRSET<br>backbone<br>zz-linker<br>mCherry<br>PIF6(1-100)<br>PAS Linker<br>6xHis-tag<br>Template for<br>pMH1452	…GGAGATATACATATGGCGCAACACGATGAAGCCGTAGACA<br>ACAAATTCAACAAAGAACAACAAAACGCGTTCTATGAGATC<br>TTACATTTACCTAACTTAAACGAAGAACAACGAAACGCCTT<br>CATCCAAAGTTTAAAAGATGACCCAAGCCAAAGCGCTAACC<br>TTTTAGCAGAAGCTAAAAAGCTAAATGATGCTCAGGCGCCG<br>AAAGTAGACAACAAATTCAACAAAGAACAACAAAACGCGTT<br>CTATGAGATCTTACATTTACCTAACTTAAACGAAGAACAAC<br>GAAACGCCTTCATCCAAAGTTTAAAAGATGACCCAAGCCAA<br>AGCGCTAACCTTTTAGCAGAAGCTAAAAAGCTAAATGATGC<br>TCAGGCGCCGAAAGTAGACGCGAATTCGAGCGGTGCAGGTA<br>GCGGTTCTGGCATGGTGAGCAAGGGCGAGGAGGATAACATG<br>GCCATCATCAAGGAGTTCATGCGCTTCAAGGTGCACATGGA<br>GGGCTCCGTGAACGGCCACGAGTTCGAGATCGAGGGCGAGG<br>GCGAGGGCCGCCCCTACGAGGGCACCCAGACCGCCAAGCTG<br>AAGGTGACCAAGGGTGGCCCCCTGCCCTTCGCCTGGGACAT<br>CCTGTCCCCTCAGTTCATGTACGGCTCCAAGGCCTACGTGA<br>AGCACCCCGCCGACATCCCCGACTACTTGAAGCTGTCCTTC<br>CCCGAGGGCTTCAAGTGGGAGCGCGTGATGAACTTCGAGGA<br>CGGCGGCGTGGTGACCGTGACCCAGGACTCCTCCCTGCAGG<br>ACGGCGAGTTCATCTACAAGGTGAAGCTGCGCGGCACCAAC<br>TTCCCCTCCGACGGCCCCGTAATGCAGAAGAAGACCATGGG

	CTGGGAGGCCTCCTCCGAGCGGATGTACCCCGAGGACGGCG<br>CCCTGAAGGGCGAGATCAAGCAGAGGCTGAAGCTGAAGGAC<br>GGCGGCCACTACGACGCTGAGGTCAAGACCACCTACAAGGC<br>CAAGAAGCCCGTGCAGCTGCCCGGCGCCTACAACGTCAACA<br>TCAAGTTGGACATCACCTCCCACAACGAGGACTACACCATC<br>GTGGAACAGTACGAACGCGCCGAGGGCCGCCACTCCACCGG<br>CGGCATGGACGAGCTGTACAAG GGCTCCGCAGGTTCTGCT<br>GGTATGATGTTCTTACCAACCGATTATTGTTGCAGGTTAAG<br>CGATCAAGAGTATATGGAGCTTGTGTTTGAGAATGGCCAGA<br>TTCTTGCAAAGGGCCAAAGATCCAACGTTTCTCTGCATAAT<br>CAACGTACCAAATCGATCATGGATTTGTATGAGGCAGAGTA<br>TAACGAGGATTTCATGAAGAGTATCATCCATGGTGGTGGTG<br>GTGCCATCACAAATCTCGGGGACACGCAGGTTGTTCCACAA<br>AGTCATGTTGCTGCTGCCCATGAAACAAACATGTTGGAAAG<br>CAATAAACATGTTGACGGCTCTTCTGCCTCTCCAGCTGCAC<br>CTGCTCCCGCTTCACCAGCGGCCCCCGCCCCTTCAGCTCCA<br>GCAGCAAGTCCGGCTGCTCCTGCGCCTGCTTCGCCTGCAGC<br>GCCCGCTCCGTCCGCACCCGCGGCCTCCCCGGCCGCCCCGG<br>CGCCGGCGAGCCCGGCGGCACCGGCCCCGTCTGCTCCTGCT<br>GCCATGATGTTCTTACCAACCGATTATTGTTGCAGGTTAAG<br>CGATCAAGAGTATATGGAGCTTGTGTTTGAGAATGGCCAGA<br>TTCTTGCAAAGGGCCAAAGATCCAACGTTTCTCTGCATAAT<br>CAACGTACCAAATCGATCATGGATTTGTATGAGGCAGAGTA

	TAACGAGGATTTCATGAAGAGTATCATCCATGGTGGTGG<br>GTGCCATCACAAATCTCGGGGACACGCAGGTTGTTCCACAA<br>AGTCATGTTGCTGCTGCCCATGAAACAAACATGTTGGAAAG<br>CAATAAACATGTTGACCATCATCACCATCACCATTAAAAGC<br>TTG...
pMH1450<br>pRSET<br>backbone<br>zz-linker<br>mEGFP<br>PIF6(1-100)<br>6xHis-tag	...GGAGATATACATATGGCGCAACACGATGAAGCCGTAGACA<br>ACAAATTCAACAAAGAACAACAAAACGCGTTCTATGAGATC<br>TTACATTTACCTAACTTAAACGAAGAACAACGAAACGCCTT<br>CATCCAAAGTTTAAAAGATGACCCAAGCCAAAGCGCTAACC<br>TTTTAGCAGAAGCTAAAAAGCTAAATGATGCTCAGGCGCCG<br>AAAGTAGACAACAAATTCAACAAAGAACAACAAAACGCGTT<br>CTATGAGATCTTACATTTACCTAACTTAAACGAAGAACAAC<br>GAAACGCCTTCATCCAAAGTTTAAAAGATGACCCAAGCCAA<br>AGCGCTAACCTTTTAGCAGAAGCTAAAAAGCTAAATGATGC<br>TCAGGCGCCGAAAGTAGACGCGAATTCGAGCGGTGCAGGTA<br>GCGGTTCTGGCATGGTGAGCAAGGGCGAGGAGCTGTTCACC<br>GGGGTGGTGCCCATCCTGGTCGAGCTGGACGGCGACGTAAA<br>CGGCCACAAGTTCAGCGTGTCCGGCGAGGGCGAGGGCGATG<br>CCACCTACGGCAAGCTGACCCTGAAGTTCATCTGCACCACC<br>GGCAAGCTGCCCGTGCCCTGGCCCACCCTCGTGACCACCCT<br>GACCTACGGCGTGCAGTGCTTCAGCCGCTACCCCGACCACA<br>TGAAGCAGCACGACTTCTTCAAGTCCGCCATGCCCGAAGGC<br>TACGTCCAGGAGCGCACCATCTTCTTCAAGGACGACGGCAA

	CTACAAGACCCGCGCCGAGGTGAAGTTCGAGGGCGACACCC<br>TGGTGAACCGCATCGAGCTGAAGGGCATCGACTTCAAGGAG<br>GACGGCAACATCCTGGGGCACAAGCTGGAGTACAACTACAA<br>CAGCCACAACGTCTATATCATGGCCGACAAGCAGAAGAACG<br>GCATCAAGGTGAACTTCAAGATCCGCCACAACATCGAGGAC<br>GGCAGCGTGCAGCTCGCCGACCACTACCAGCAGAACACCCC<br>CATCGGCGACGGCCCCGTGCTGCTGCCCGACAACCACTACC<br>TGAGCACCCAGTCCAAGCTGAGCAAAGACCCCAACGAGAAG<br>CGCGATCACATGGTCCTGCTGGAGTTCGTGACCGCCGCCGG<br>GATCACTCTCGGCATGGACGAGCTGTACAAGGGCTCCGCAG<br>GTTCTGCTGGTATGATGTTCTTACCAACCGATTATTGTTGC<br>AGGTTAAGCGATCAAGAGTATATGGAGCTTGTGTTTGAGAA<br>TGGCCAGATTCTTGCAAAGGGCCAAAGATCCAACGTTTCTC<br>TGCATAATCAACGTACCAAATCGATCATGGATTTGTATGAG<br>GCAGAGTATAACGAGGATTTCATGAAGAGTATCATCCATGG<br>TGGTGGTGGTGCCATCACAAATCTCGGGGACACGCAGGTTG<br>TTCCACAAAGTCATGTTGCTGCTGCCCATGAAACAAACATG<br>TTGGAAAGCAATAAACATGTTGACCATCATCACCATCACCA<br>TTAAAAGCTTG…
pMH1451<br><br>pRSET<br><br>backbone	…GGAGATATACATATGGCGCAACACGATGAAGCCGTAGACA<br>ACAAATTCAACAAAGAACAACAAAACGCGTTCTATGAGATC<br>TTACATTTACCTAACTTAAACGAAGAACAACGAAACGCCTT<br>CATCCAAAGTTTAAAAGATGACCCAAGCCAAAGCGCTAACC

zz-linker<br><br>mEGFP<br><br>PIF6(1-100)<br><br>AviTag<br><br>6xHis-tag	TTTTAGCAGAAGCTAAAAAGCTAAATGATGCTCAGGCGCCG<br>AAAGTAGACAACAAATTCAACAAAGAACAACAAAACGCGTT<br>CTATGAGATCTTACATTTACCTAACTTAAACGAAGAACAAC<br>GAAACGCCTTCATCCAAAGTTTAAAAGATGACCCAAGCCAA<br>AGCGCTAACCTTTTAGCAGAAGCTAAAAAGCTAAATGATGC<br>TCAGGCGCCGAAAGTAGACGCGAATTCGAGCGGTGCAGGTA<br>GCGGTTCTGGCATGGTGAGCAAGGGCGAGGAGCTGTTCACC<br>GGGGTGGTGCCCATCCTGGTCGAGCTGGACGGCGACGTAAA<br>CGGCCACAAGTTCAGCGTGTCCGGCGAGGGCGAGGGCGATG<br>CCACCTACGGCAAGCTGACCCTGAAGTTCATCTGCACCACC<br>GGCAAGCTGCCCGTGCCCTGGCCCACCCTCGTGACCACCCT<br>GACCTACGGCGTGCAGTGCTTCAGCCGCTACCCCGACCACA<br>TGAAGCAGCACGACTTCTTCAAGTCCGCCATGCCCGAAGGC<br>TACGTCCAGGAGCGCACCATCTTCTTCAAGGACGACGGCAA<br>CTACAAGACCCGCGCCGAGGTGAAGTTCGAGGGCGACACCC<br>TGGTGAACCGCATCGAGCTGAAGGGCATCGACTTCAAGGAG<br>GACGGCAACATCCTGGGGCACAAGCTGGAGTACAACTACAA<br>CAGCCACAACGTCTATATCATGGCCGACAAGCAGAAGAACG<br>GCATCAAGGTGAACTTCAAGATCCGCCACAACATCGAGGAC<br>GGCAGCGTGCAGCTCGCCGACCACTACCAGCAGAACACCCC<br>CATCGGCGACGGCCCCGTGCTGCTGCCCGACAACCACTACC<br>TGAGCACCCAGTCCAAGCTGAGCAAAGACCCCAACGAGAAG<br>CGCGATCACATGGTCCTGCTGGAGTTCGTGACCGCCGCCGG

	GATCACTCTCGGCATGGACGAGCTGTACAAGGGCTCCGCAG<br>GTTCTGCTGGTATGATGTTCTTACCAACCGATTATTGTTGC<br>AGGTTAAGCGATCAAGAGTATATGGAGCTTGTGTTTGAGAA<br>TGGCCAGATTCTTGCAAAGGGCCAAAGATCCAACGTTTCTC<br>TGCATAATCAACGTACCAAATCGATCATGGATTTGTATGAG<br>GCAGAGTATAACGAGGATTTCATGAAGAGTATCATCCATGG<br>TGGTGGTGGTGCCATCACAAATCTCGGGGACACGCAGGTTG<br>TTCCACAAAGTCATGTTGCTGCTGCCCATGAAACAAACATG<br>TTGGAAAGCAATAAACATGTTGACGGTCTGAACGACATCTT<br>CGAAGCTCAGAAAATCGAATGGCACGAACATCATCACCATC<br>ACCATTAAAAGCTTG...
pMH1452<br><br>pRSET<br>backbone<br><br>zz-linker<br><br>mEFGP<br><br>PIF6(1-100)<br><br>PAS Linker<br><br>6xHis-tag	...GGAGATATACATATGGCGCAACACGATGAAGCCGTAGACA<br>ACAAATTCAACAAAGAACAACAAAACGCGTTCTATGAGATC<br>TTACATTTACCTAACTTAAACGAAGAACAACGAAACGCCTT<br>CATCCAAAGTTTAAAAGATGACCCAAGCCAAAGCGCTAACC<br>TTTTAGCAGAAGCTAAAAAGCTAAATGATGCTCAGGCGCCG<br>AAAGTAGACAACAAATTCAACAAAGAACAACAAAACGCGTT<br>CTATGAGATCTTACATTTACCTAACTTAAACGAAGAACAAC<br>GAAACGCCTTCATCCAAAGTTTAAAAGATGACCCAAGCCAA<br>AGCGCTAACCTTTTAGCAGAAGCTAAAAAGCTAAATGATGC<br>TCAGGCGCCGAAAGTAGACGCGAATTCGAGCGGTGCAGGTA<br>GCGGTTCTGGCATGGTGAGCAAGGGCGAGGAGCTGTTCACC<br>GGGGTGGTGCCCATCCTGGTCGAGCTGGACGGCGACGTAAA

	CGGCCACAAGTTCAGCGTGTCCGGCGAGGGCGAGGGCGATG<br>CCACCTACGGCAAGCTGACCCTGAAGTTCATCTGCACCACC<br>GGCAAGCTGCCCGTGCCCTGGCCCACCCTCGTGACCACCCT<br>GACCTACGGCGTGCAGTGCTTCAGCCGCTACCCCGACCACA<br>TGAAGCAGCACGACTTCTTCAAGTCCGCCATGCCCGAAGGC<br>TACGTCCAGGAGCGCACCATCTTCTTCAAGGACGACGGCAA<br>CTACAAGACCCGCGCCGAGGTGAAGTTCGAGGGCGACACCC<br>TGGTGAACCGCATCGAGCTGAAGGGCATCGACTTCAAGGAG<br>GACGGCAACATCCTGGGGCACAAGCTGGAGTACAACTACAA<br>CAGCCACAACGTCTATATCATGGCCGACAAGCAGAAGAACG<br>GCATCAAGGTGAACTTCAAGATCCGCCACAACATCGAGGAC<br>GGCAGCGTGCAGCTCGCCGACCACTACCAGCAGAACACCCC<br>CATCGGCGACGGCCCCGTGCTGCTGCCCGACAACCACTACC<br>TGAGCACCCAGTCCAAGCTGAGCAAAGACCCCAACGAGAAG<br>CGCGATCACATGGTCCTGCTGGAGTTCGTGACCGCCGCCGG<br>GATCACTCTCGGCATGGACGAGCTGTACAAGGGCTCCGCAG<br>GTTCTGCTGGTATGATGTTCTTACCAACCGATTATTGTTGC<br>AGGTTAAGCGATCAAGAGTATATGGAGCTTGTGTTTGAGAA<br>TGGCCAGATTCTTGCAAAGGGCCAAAGATCCAACGTTTCTC<br>TGCATAATCAACGTACCAAATCGATCATGGATTTGTATGAG<br>GCAGAGTATAACGAGGATTTCATGAAGAGTATCATCCATGG<br>TGGTGGTGGTGCCATCACAAATCTCGGGGACACGCAGGTTG<br>TTCCACAAAGTCATGTTGCTGCTGCCCATGAAACAAACATG

	TTGGAAAGCAATAAACATGTTGACGGCTCTTCTGCCTCTCC<br>AGCTGCACCTGCTCCCGCTTCACCAGCGGCCCCCGCCCCTT<br>CAGCTCCAGCAGCAAGTCCGGCTGCTCCTGCGCCTGCTTCG<br>CCTGCAGCGCCCGCTCCGTCCGCACCCGCGGCCTCCCCGGC<br>CGCCCCGGCGCCGGCGAGCCCGGCGGCACCGGCCCCGTCTG<br>CTCCTGCTGCCATGATGTTCTTACCAACCGATTATTGTTGC<br>AGGTTAAGCGATCAAGAGTATATGGAGCTTGTGTTTGAGAA<br>TGGCCAGATTCTTGCAAAGGGCCAAAGATCCAACGTTTCTC<br>TGCATAATCAACGTACCAAATCGATCATGGATTTGTATGAG<br>GCAGAGTATAACGAGGATTTCATGAAGAGTATCATCCATGG<br>TGGTGGTGGTGCCATCACAAATCTCGGGGACACGCAGGTTG<br>TTCCACAAAGTCATGTTGCTGCTGCCCATGAAACAAACATG<br>TTGGAAAGCAATAAACATGTTGACCATCATCACCATCACCA<br>TTTAAAAGCTTG…
pKJ10<br><br>pRSET<br><br>backbone<br><br>6xHis-tag<br><br>mCherry	…GGAGATATACATATGCATCATCACCATCACCATGTGAGCA<br>AGGGCGAGGAGGATAACATGGCCATCATCAAGGAGTTCATG<br>CGCTTCAAGGTGCACATGGAGGGCTCCGTGAACGGCCACGA<br>GTTCGAGATCGAGGGCGAGGGCGAGGGCCGCCCCTACGAGG<br>GCACCCAGACCGCCAAGCTGAAGGTGACCAAGGGTGGCCCC<br>CTGCCCTTCGCCTGGGACATCCTGTCCCCTCAGTTCATGTA<br>CGGCTCCAAGGCCTACGTGAAGCACCCCGCCGACATCCCCG<br>ACTACTTGAAGCTGTCCTTCCCCGAGGGCTTCAAGTGGGAG<br>CGCGTGATGAACTTCGAGGACGGCGGCGTGGTGACCGTGAC

	CCAGGACTCCTCCCTGCAGGACGGCGAGTTCATCTACAAGG<br>TGAAGCTGCGCGGCACCAACTTCCCCTCCGACGGCCCCGTA<br>ATGCAGAAGAAGACCATGGGCTGGGAGGCCTCCTCCGAGCG<br>GATGTACCCCGAGGACGGCGCCCTGAAGGGCGAGATCAAGC<br>AGAGGCTGAAGCTGAAGGACGGCGGCCACTACGACGCTGAG<br>GTCAAGACCACCTACAAGGCCAAGAAGCCCGTGCAGCTGCC<br>CGGCGCCTACAACGTCAACATCAAGTTGGACATCACCTCCC<br>ACAACGAGGACTACACCATCGTGGAACAGTACGAACGCGCC<br>GAGGGCCGCCACTCCACCGGCGGCATGGACGAGCTGTACAA<br>GTAATAAAAGCTTG…
pHB111(35)	mEGFP-PIF6(1-100)-His6
pMH1105(36)	PhyB(1-651)-AviTag-His6
pWW873(37)	GyrB-His6

Auxiliary Material

Data S1: Measurement data from Figure 3A

Data S2: Measurement data from Figure S1

Movie S1. Video of the OptoAssay procedure

Movie S2. Video demonstrating the kinetics and reversibility of the PhyB/PIF system. Scale bar: 50 µm
